# A simplified synthetic community rescues *Astragalus mongholicus* from root rot disease by activating plant-induced systemic resistance

**DOI:** 10.1186/s40168-021-01169-9

**Published:** 2021-11-04

**Authors:** Zhefei Li, Xiaoli Bai, Shuo Jiao, Yanmei Li, Peirong Li, Yan Yang, Hui Zhang, Gehong Wei

**Affiliations:** State key Laboratory of Crop Stress Biology in Arid Areas, Shaanxi Key Laboratory of Agricultural and Environmental Microbiology, College of Life Science, Northwest A&F University, Yangling, 712100 Shaanxi China

**Keywords:** Low-abundance bacteria, High-abundance bacteria, Synthetic community, Community simplification, Root rot disease

## Abstract

**Background:**

Plant health and growth are negatively affected by pathogen invasion; however, plants can dynamically modulate their rhizosphere microbiome and adapt to such biotic stresses. Although plant-recruited protective microbes can be assembled into synthetic communities for application in the control of plant disease, rhizosphere microbial communities commonly contain some taxa at low abundance. The roles of low-abundance microbes in synthetic communities remain unclear; it is also unclear whether all the microbes enriched by plants can enhance host adaptation to the environment. Here, we assembled a synthetic community with a disease resistance function based on differential analysis of root-associated bacterial community composition. We further simplified the synthetic community and investigated the roles of low-abundance bacteria in the control of *Astragalus mongholicus* root rot disease by a simple synthetic community.

**Results:**

*Fusarium oxysporum* infection reduced bacterial Shannon diversity and significantly affected the bacterial community composition in the rhizosphere and roots of *Astragalus mongholicus*. Under fungal pathogen challenge, *Astragalus mongholicus* recruited some beneficial bacteria such as *Stenotrophomonas*, *Achromobacter*, *Pseudomonas*, and *Flavobacterium* to the rhizosphere and roots. We constructed a disease-resistant bacterial community containing 10 high- and three low-abundance bacteria enriched in diseased roots. After the joint selection of plants and pathogens, the complex synthetic community was further simplified into a four-species community composed of three high-abundance bacteria (*Stenotrophomonas* sp., *Rhizobium* sp., *Ochrobactrum* sp.) and one low-abundance bacterium (*Advenella* sp.). Notably, a simple community containing these four strains and a thirteen-species community had similar effects on the control root rot disease. Furthermore, the simple community protected plants via a synergistic effect of highly abundant bacteria inhibiting fungal pathogen growth and less abundant bacteria activating plant-induced systemic resistance.

**Conclusions:**

Our findings suggest that bacteria with low abundance play an important role in synthetic communities and that only a few bacterial taxa enriched in diseased roots are associated with disease resistance. Therefore, the construction and simplification of synthetic communities found in the present study could be a strategy employed by plants to adapt to environmental stress.

Video abstract

**Supplementary Information:**

The online version contains supplementary material available at 10.1186/s40168-021-01169-9.

## Background

The rhizosphere is known as the soil region surrounding the plant roots as well as the reservoir of soil microorganisms. It hosts a complex assemblage of microbes, including bacteria, fungi, and archaea, which collectively influence plant performance [1]. A key role of the soil microbiota is to protect plant roots and leaves from pathogens, nematodes, and pests [2–4]. Consequently, the diversity and abundance of beneficial microbes, such as plant growth–promoting bacteria (PGPB) and arbuscular mycorrhizal fungi (AMF), considerably influence plant health [5, 6].

Monoculture systems and excessive chemical fertilizer application alter soil physicochemical properties, which, in turn, influence rhizosphere microbial community structure [7–9]. The reduction in beneficial soil microbiota could lead to the accumulation of pathogens in the rhizosphere, which would promote soil-borne disease spreading in crops via negative plant–soil feedback over the long term [10]. Notably, continuous cultivation of a susceptible crop could induce the suppression of soil pathogens, such as *Fusarium oxysporum*, *Pythium ultimum*, and *Rhizoctonia solani*, following several severe disease outbreaks [11–14], so that plants may not exhibit disease symptoms despite pathogen presence in the disease-suppressive soil. In addition, soil disease-suppressive capacity could be augmented by soil suspension inoculation, and disease-suppressive soil can also be converted into susceptible soil by sterilization [15]. Such phenomena attribute to the enrichment of specific microorganisms in the rhizosphere of plant roots, as these microorganisms may participate in the resistance to the invasion of pathogens [2]. Under the induction of environmental factors, the physiological status and metabolic pathway of plants changed, which influences the proportions and compositions of rhizodeposits, including root exudates and complex organic compounds resulting from sloughed-off root tissues [16]. Such rhizodeposits released by roots attract specific microorganisms from the soil to colonize the rhizosphere and roots. The microorganisms then assemble into root-associated microbial communities and lead to variations in the diversity of microbial communities in the rhizosphere [17]. However, plants are exposed to various biotic and abiotic stress factors simultaneously in the course of growth under natural conditions, and microbes with different ecological functions are enriched in the rhizosphere and roots. Even under plant pathogen infestation conditions, the enriched microorganisms are not all associated with disease resistance. Consequently, the assembled microbial communities are not able to suppress disease when the plants are initially infected by pathogens; however, root-associated microbial communities could suppress diseases after successive pathogen infestation events.

Plants recruit protective microorganisms by regulating the production of root exudates through defense-related signaling molecules such as salicylic acid (SA) and jasmonic acid (JA) [16]. Previous studies have mainly focused on isolating and screening antagonists in the rhizosphere or roots that directly inhibit pathogen growth for potential application in plant disease control [17, 18]. Over the last decade, however, multiomics sequencing technologies have been extensively applied in plant microbiome studies, and they have revealed that complex microbial community assemblages in the rhizosphere rather than single microbial strains protect plants from pathogen infection [19, 20]. In attempts to mimic natural disease-suppressive soil conditions, the potential of artificial synthetic communities composed of diverse bacteria to prevent soil-borne disease has been explored. For example, a synthetic microbial community consisting of three bacterial species, including *Xanthomonas* sp., *Stenotrophomonas* sp., and *Microbacterium* sp., could synergistically control downy mildew in *Arabidopsis thaliana* [16]. Another study demonstrated that a synthetic community consisting of 38 bacterial species could influence the immune activity of *A. thaliana* [21]. However, the structural and functional stability of such synthetic communities are influenced considerably by microbial species, which are further influenced by the host species and genotypes, in addition to biotic and abiotic stress factors from the environment [22–25]. Consequently, determining which microbial strains in an introduced community can survive and persist in interactions among bacteria, plants, and pathogens is essential for the assembly of synthetic microbial communities with disease resistance potential.

In addition to microbial diversity, the abundance of key species could influence microbial community function. Although thousands of distinct bacterial species are found in the soil, their proportions vary extensively [26]. Numerous high-throughput sequencing studies have revealed that the relative abundances of a small number of microbial taxa are greater than 1%, while a large proportion of microorganisms that belong to rare taxa have relative abundances lower than 0.1% [27]. Generally, high-abundance microbes have important ecological functions, while low-abundance taxa are considered seed banks and exhibit low metabolic activity in the soil [28]. However, some studies have demonstrated that microorganisms with low abundance have diverse metabolic functions and participate in soil nutrient transformation, redox reactions, and bioremediation activities [29–32]. Nevertheless, no evidence has shown whether low-abundance microorganisms in synthetic bacterial communities play an important role in disease suppression.

*Astragalus membranaceus* Bge. var. *mongholicus* (Bge.) Hsiao (hereafter, *Astragalus*), a perennial herbaceous leguminous plant, is one of the most important traditional Chinese herbs. It is also an important cash crop in Gansu, Shanxi, Inner Mongolia, and other provinces in China [33]. Long-term monocropping leads to a high incidence of *Astragalus* root rot. In the present study, we studied microbial community responses to the *Fusarium oxysporum* infection and investigated (1) how plant-associated microbial communities respond to *F. oxysporum* infection, (2) whether enrichment with bacteria with low relative abundance could confer root rot disease prevention functions in synthetic communities, and (3) whether simple synthetic bacterial communities have roles similar to those of complex synthetic communities.

To address the questions above, we first analyzed differences in root-associated microbial community structure between healthy and diseased plants. Afterward, we constructed a synthetic community (SCI) consisting of 10 high-abundance and three low-abundance bacteria enriched in diseased roots. In addition, we selected nine bacterial strains (two strains for *Bacillus*) with decreased abundance and randomly selected four strains in diseased roots to construct synthetic community II (SCII), which served as the control. Furthermore, using a combination of PacBio sequencing and plant selection, we simplified the complex synthetic community (SCI) into a simple four-species synthetic community (SCIII) that conferred root rot disease resistance. Finally, we investigated the separate and joint effects of the four bacteria in SCIII on *F. oxysporum* growth, plant growth promotion, host plant–induced systemic resistance (ISR) activation, and root rot control.

## Materials and methods

### Sample preparation and soil physicochemical property measurement

The samples used in this work were collected from three medicinal plant fields in Tanchang County, Gansu Province, China (N34° 15.123’; E104° 09.727’**)**. The soil organic matter, total nitrogen, total phosphorus, total potassium concentrations, and pH were 2.76 g/kg, 1.75 g/kg, 1.31 g/kg, 10.86 g/kg, and 8.17, respectively. The fields had a 4-year history of potato and *Astragalus* rotation before the samples were collected. In March 2019, 1-year-old *Astragalus* seedlings were transplanted to the fields, and 24 plants (12 healthy + 12 diseased plants) were uprooted randomly with shovels when root rot disease was determined to be severe in May 2019. Roots were shaken vigorously to remove loose soil and the 1–2-mm–thick soil layer surrounding the root was defined as the rhizosphere soil. To collect the rhizosphere soil, the roots were transferred into tubes containing 25 mL of 1× phosphate buffer. The tubes were inverted 4–5 times and vortexed for 5 min, and then roots were removed. Afterward, the tubes were centrifuged at 2500 × *g* for 5 min. The supernatant was discarded, and the remaining material was centrifuged at 2500 × *g* for 5 min. After collecting the rhizosphere soil, the remaining roots were surface-disinfected with 75% ethanol for 1 min and then 1% sodium hypochlorite for 30 s. After that, the roots were rinsed 10 times with sterile water (Fig. s1). In total, 72 samples were obtained from bulk soil, rhizosphere soil, and roots (3 compartments × 3 fields × 4 repetitions × 2 health states). All the roots and soil samples were stored at − 80 °C before DNA isolation.

### DNA extraction, amplification, and sequencing

All the 72 samples were divided into two groups: the first group was transported to the laboratory at room temperature for microbial isolation, and the other group was frozen and instantly sent to the laboratory for DNA extraction. Total DNA was extracted from 0.5 g of soil or root using a FastDNA®SPIN Kit (MP Biomedicals, Solon, USA), according to the manufacturer’s protocol. The barcoded primers 515F (GTGCCAGCMGCCGCGG) /907R (CCGTCAATTCMTTTRAGT) were used to amplify the V4–V5 region of bacterial 16S rRNA, and the primers ITS1F (CTTGGTCATTTAGAGGAAGTAA)/ITS1R (GCTGCGTTCTT CATCGATGC) were used to amplify the ITS1 regions of fungi. The purified PCR products were sequenced on the Illumina MiSeq PE300 platform at Major Biomedical Technology Co., Ltd (Shanghai, China). Raw data were assembled and quality-filtered according to Caporaso et al. [34], and chimeric sequences were removed using the UCHIME tool in USEARCH [35]. The sequences matching the mitochondria and chloroplast were also removed [36], and the remaining effective sequences were clustered into operational taxonomic units (OTUs) at 97% similarity [37].

### Isolation of plant-associated bacteria and pathogenic fungi from field-grown plants

Field-grown *Astragalus* at the vegetative stage displaying symptoms of root rot disease were used for the isolation of fungal pathogens according to Schuck et al. [38] with minor modifications (Supplementary Materials). Plant-associated bacteria were isolated as described in [39]. Bacterial 16S rDNA and fungal 18S rDNA were amplified and sequenced at Sangon Biotech Co., Ltd (Shanghai, China). Sequence alignment was performed on the NCBI website, and each microbial species was preserved in 1 mL of 30% glycerol (v/v) at − 80 °C. The healthy plants were then reinoculated with the potential pathogens, and the pathogenic capacity of each fungus was evaluated.

### Assembly and simplification of synthetic communities

Based on the root-associated bacterial community composition and diversity in diseased plants, some bacteria that were significantly enriched and depleted in the rhizosphere or roots were selected as candidate strains for bacterial community construction. Following measurement of the P dissolution, K dissolution, indole acetic acid (IAA) production, and *F. oxysporum* antagonism by the candidate bacteria, we selected 13 bacterial species with plant growth promotion or pathogenic fungal growth inhibition capacity, which were obviously enriched in *Astragalus* diseased roots (Table s1), and equal volumes of each strain (~ 10^8^ cells/mL) were mixed to establish synthetic community I (SCI).

The abundant genera (relative abundance > 0.1%) accounted for 98.02% of all OTUs. Therefore, we classified the genera with abundance > 0.1% and abundance < 0.1% as high-abundance bacteria and low-abundance bacteria, respectively [27]. Consequently, SCI contained 10 high- and three low-abundance bacteria. In addition, synthetic community II (SCII) was assembled using nine depleted bacteria and four random bacteria found in diseased roots (Table s1). Then, the effects of synthetic communities on the incidence of root rot disease were investigated using pot experiments, and sterile water was used as a negative control. In addition, the effects of synthetic communities on root length, shoot length, plant fresh weight, and plant dry weight were also determined. For detailed methods, see Supplementary Materials.

To determine which bacteria from the synthetic bacterial communities were retained and performed functions in the rhizosphere or roots during interactions among plants, *F. oxysporum*, and bacterial communities, we inoculated 7-day-old *Astragalus* seedlings with SCI and grew them in a greenhouse for 30 days. DNA extraction and near full-length 16S rRNA library preparation were performed as described previously [40]. Sequencing of near full-length 16S rRNA amplicons and the assembly of reads were performed on the PacBio platform at Novogene Technology Co., Ltd (Beijing, China). A four-species community (SCIII) was constructed using strains that could colonize the rhizosphere or roots, based on the 16S rRNA full-length sequencing results.

### Disease control by synthetic bacterial communities

The capacity of synthetic bacterial communities to control *Astragalus* root rot was evaluated *in vivo*. A planting bag was filled with 500 g of substrate (soil:vermiculite:peat = 3:2:1, v/v) for autoclaving. Subsequently, each bag was inoculated with 10 mL of SCI, SCII, or SCIII; sealed; and incubated for 7 days. Soil medium supplemented with the same volume of sterile water was used as the control, and each treatment contained 10 bags. *Astragalus* seeds were surface-sterilized with 75% ethanol for 3 min and 3% sodium hypochlorite solution for 8 min, followed by six-time rinsing with deionized distilled water. Sterilized seeds were germinated on wet filter paper in Petri dishes for 48 h. Ten germinated seedlings were sown in a planting bag. After 7 days, the seedlings were thinned, and five uniform plants were maintained. The remaining plants were inoculated again with 10 mL of the synthetic communities, and the plants were grown continuously for 5 days before adding a 10-mL spore suspension of *F. oxysporum* (~ 10^5^ spores per milliliter). All the plants were grown in a greenhouse at 25 °C 16 h light/8 h darkness). Root rot incidence and plant mortality were assessed every 3 days. The antagonistic activity of SCIII against pathogenic fungi was examined by assessing fungal growth inhibition using a confrontation bioassay.

### Induced systemic resistance assay

Four-week-old plants were inoculated with 10 mL of sterile water, *Stenotrophomonas* sp*.*, *Rhizobium* sp., *Advenella* sp., *Ochrobactrum* sp., and different synthetic communities, as mentioned above. Plants were grown in planting bags placed in a greenhouse for 10 days. Plant samples were harvested at 8, 24, 72, 120, and 168 h after inoculation with different bacterial or synthetic communities. Subsequently, phenylalanine ammonia lyase (PAL), polyphenol oxidase (PPO), peroxidase (POD), lipoxygenase (LOX), chitinase activity, and jasmonic acid (JA) contents were measured. For detailed methods, see Supplemental Materials.

### Effect of bacteria of different abundance in synthetic communities on plant health and ISR

To verify that *Ochrobactrum* sp. and *Advenella* sp. from SCIII could perform functions at low abundance, *Stenotrophomonas* sp*.*, *Rhizobium* sp., *Ochrobactrum* sp., and *Advenella* sp. was mixed in 10:10:1:1 and 100:100:1:1 proportions. Additionally, *Stenotrophomonas*:*Rhizobium*:*Ochrobactrum*:*Advenella* at 600:200:10:1 was also included according to the 16S amplicon sequence (Table s4). Four-week-old plants were inoculated with 10 mL of different bacterial mixtures, and a similar volume of sterile water was used as the control. Then, the root rot incidence, PAL, PPO, POD, LOX, chitinase activities, and JA content were measured as mentioned above.

### Statistical analyses

All statistical analyses were performed in the R environment (version: V3.6.0, http://www.r-project.org/). Alpha diversity indices, including the Pielou index, Shannon index, Simpson index, and Chao1 index, were analyzed using the “vegan” package in R. The results were visualized using the “ggplot2” package. The alpha diversity indices of different samples were analyzed using analysis of variance (ANOVA). Permutational multivariate analysis of variance (PERMANOVA) with 999 permutations was used to evaluate the effect of *F. oxysporum* infection on bacterial community structure using the Adonis function (vegan package) in R [41]. Differences in the abundance and enrichment of bacterial genera were examined using Kruskal–Wallis tests, and ternary plots were created using the “vcd” package and visualized by “ggplot2” in R. Spearman correlations were calculated between microbial and pathogen parameters using the “ggcor” package in R [42]. The enriched OTUs and depleted OTUs in roots between the healthy and diseased roots were analyzed using the “EdgeR” package in R [43]. The distinct and shared bacteria were analyzed with Venn diagrams using the “VennDiagram” package in R V3.6.0. PICRUSt software was used to predict KEGG ortholog functional profiles of bacterial communities in rhizospheres and roots using the 16S rRNA sequences [44]. Phylogenetic trees of bacteria isolated from rhizospheres and roots were drawn using MEGA 6, based on 16S rDNA.

## Results

### Isolation and verification of fungal pathogens from diseased plants

**Fig. 1:**
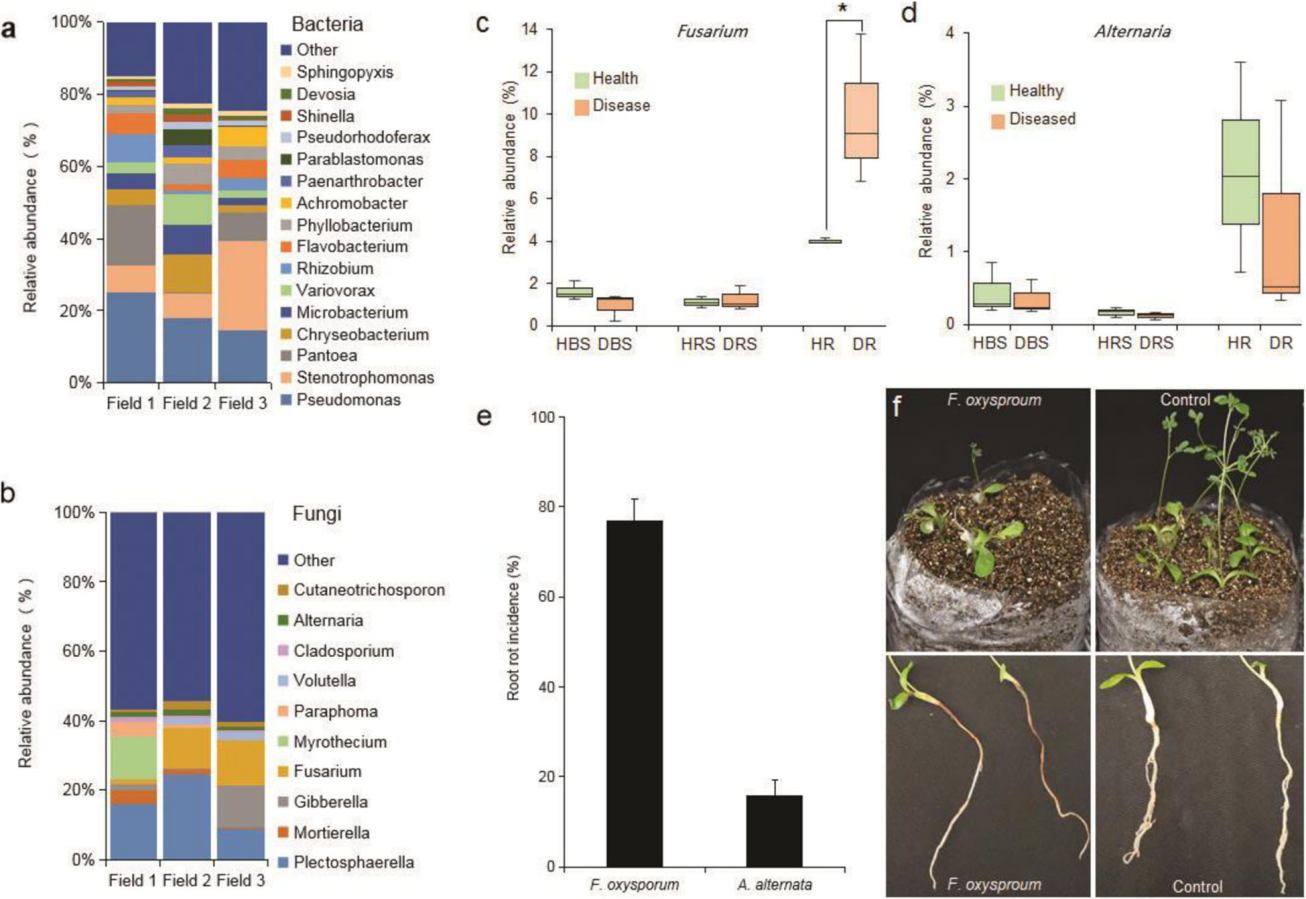
Microbial community composition in diseased roots and pathogenic capacity of potential fungal pathogens. **a** Dominant bacterial genera in field-grown plants with root rot symptoms. **b** Dominant fungal genera in field-grown plants with root rot symptoms. Two fungal genera (*Fusarium* and *Alternaria*) related to root rot disease were observed. **c** and **d** Relative abundance of *Fusarium* and *Alternaria* in healthy and diseased roots, rhizosphere, and bulk soil; *HBS*, healthy bulk soil; *DBS*, diseased bulk soil; *HRS*, healthy rhizosphere; *DRS*, diseased rhizosphere; *HR*, healthy root; *DR*, diseased root. **e** Root rot disease incidence in *Astragalus* with fungal pathogen infection. **f** Shoot and root symptoms of plants with and without *F. oxysporum* infection

As illustrated in Fig. 1, *Pseudomonas*, *Stenotrophomonas*, *Pantoea*, and *Chryseobacterium* had relatively high abundance in the bacterial communities of diseased roots, while *Plectosphaerella*, *Cephalotrichum*, and *Fusarium* had relatively high abundance in the fungal communities of diseased roots. According to previous studies, *Ralstonia solanacearum*, *Cylindrocarpon destructans*, *Fusarium oxysporum*, *Fusarium solani*, *Alternaria* sp., and *Phytophthora incognitae* are the potential pathogens causing root rot [45–47]. However, no potentially pathogenic bacteria that could be clustered into known genera from diseased roots were observed among the highly abundant OTUs (Fig. 1a). In contrast, *Fusarium* (8.79%) and *Alternaria* (1.30%) accounted for a relatively high abundance of fungi in diseased roots (Fig. 1b). *Fusarium* was significantly enriched in the roots of the diseased plants; however, there was no difference in *Alternaria* abundance between the healthy and diseased roots (Fig. 1c and d). In addition, 53 culturable fungi, including *F. oxysporum*, *F. solani*, and *Alternaria alternata*, were isolated from diseased *Astragalus*. According to the pot experiment results, only *F. oxysporum* caused high disease incidence, and plants displayed typical root rot symptoms: curly leaves, short plants, and brown and decaying roots (Fig. 1e and f). Therefore, this fungus was used as an indicator in subsequent experiments.

### Effect of fungal pathogens on the structure of root-associated bacterial communities

According to the 16S amplicon sequencing results, a total of 2,287,677 high-quality bacterial reads were obtained. After removing the low-quality and plant-derived reads, the remaining reads were clustered into 7547 bacterial OTUs at ≥ 97% sequence identity. The majority of the bacterial OTUs discovered in the healthy and diseased roots were also present in the rhizosphere. However, a considerable proportion of the bacteria was observed solely in the diseased rhizosphere and root (9.2% and 0.6%, respectively), although a majority of the OTUs associated with diseased plants were also present in the rhizosphere and roots of healthy plants (Fig. s2).

**Fig. 2:**
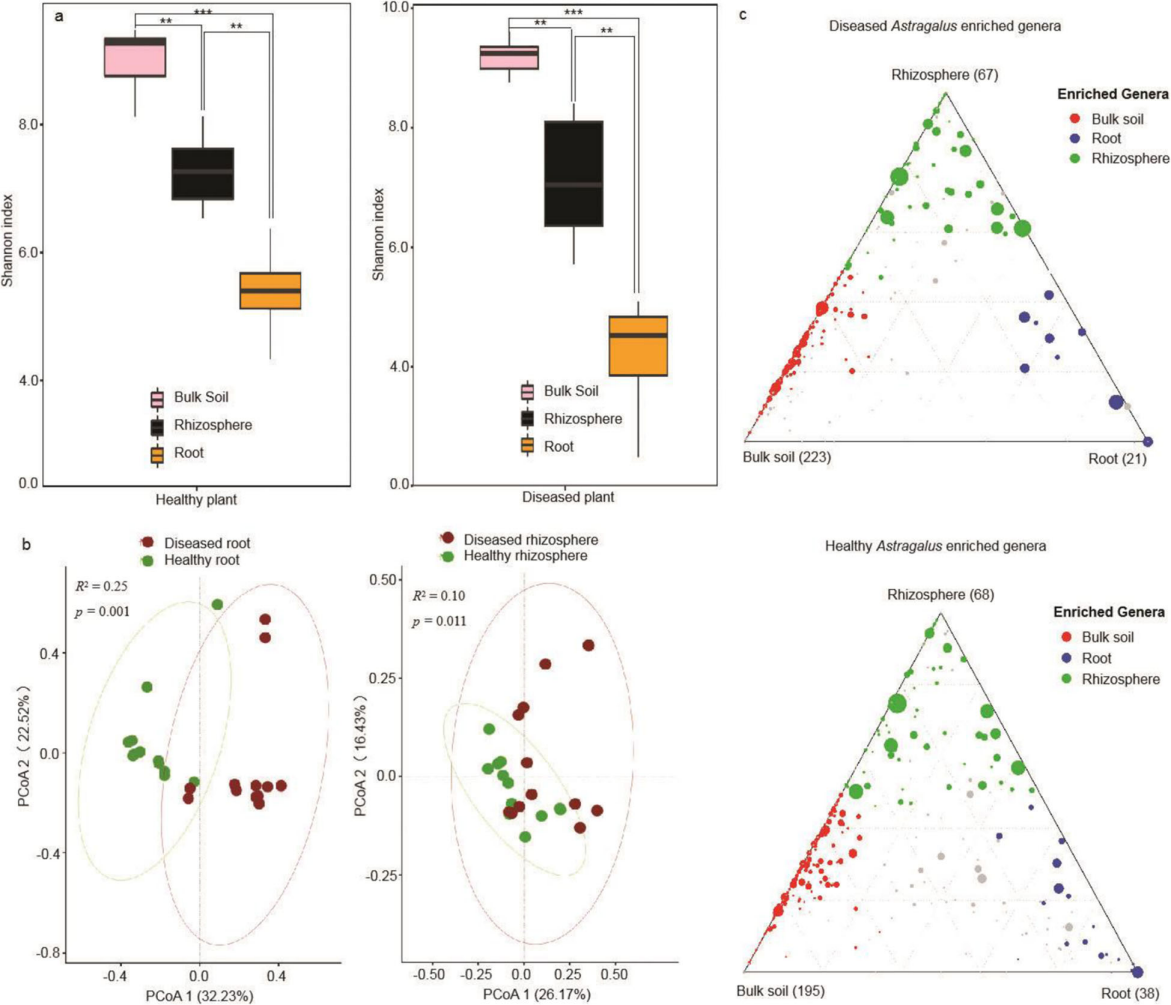
Effect of *F. oxysporum* on bacterial community structure in the rhizosphere and roots. **a** Shannon diversity of bacteria in bulk soil, rhizosphere, and roots of healthy and diseased plants. Asterisks indicate statistically significant (*P < 0.05; **P < 0.01) differences based on analysis of variance with Dunn’s multiple comparison test. **b** Principal coordinates analysis (PCoA) of root microbial communities based on Bray-Curtis distance. Fungal pathogen infection altered the bacterial community structure. **c** Differences in the abundance and enrichment of bacterial genera in three compartments of healthy and diseased plants analyzed using the Kruskal–Wallis test. The enriched bacterial genera in the diseased roots and the rhizosphere were different from those in the healthy samples

As shown in Fig. 2a, the Shannon α-diversity of the bacterial community decreased gradually from bulk soil to roots in the presence and absence of *F. oxysporum* (healthy roots: *F*_2, 33_ = 90.96, *P* < 0.001; diseased root: *F*_2, 33_ = 84.78, *P* < 0.001), which indicated that the plants had a strong selective effect on bacterial colonization in roots. In addition, the invasion of *F. oxysporum* decreased the Shannon α-diversity of bacterial communities in roots without influencing the α-diversity in bulk soil and rhizosphere (rhizosphere: *F*_1, 22_ = 0.48, *P* < 0.50; root: *F*_1, 22_ = 9.71, *P* = 0.005) (Fig. s3 and Table s2). Principal coordinate analysis (PCoA) based on Bray-Curtis revealed significant differences in the composition of bacterial communities in roots and rhizospheres between healthy and diseased plants. In addition, the difference in endophytic community composition between the healthy and diseased roots was greater than that in the rhizosphere (root: *R*^2^ = 0.25, *P* = 0.001; rhizosphere: *R*^2^ = 0.10, *P* = 0.011) (Fig. 2b). Ternary plots also showed that many bacterial OTUs enriched in diseased roots and rhizospheres were significantly different from those in the healthy plants (Fig. 2c). However, no differences were observed in bulk soil samples. The results suggested that the plants recruited certain microbes to colonize the rhizosphere and roots under *F. oxysporum* stress.

### Plant-associated microbial community composition

**Fig. 3:**
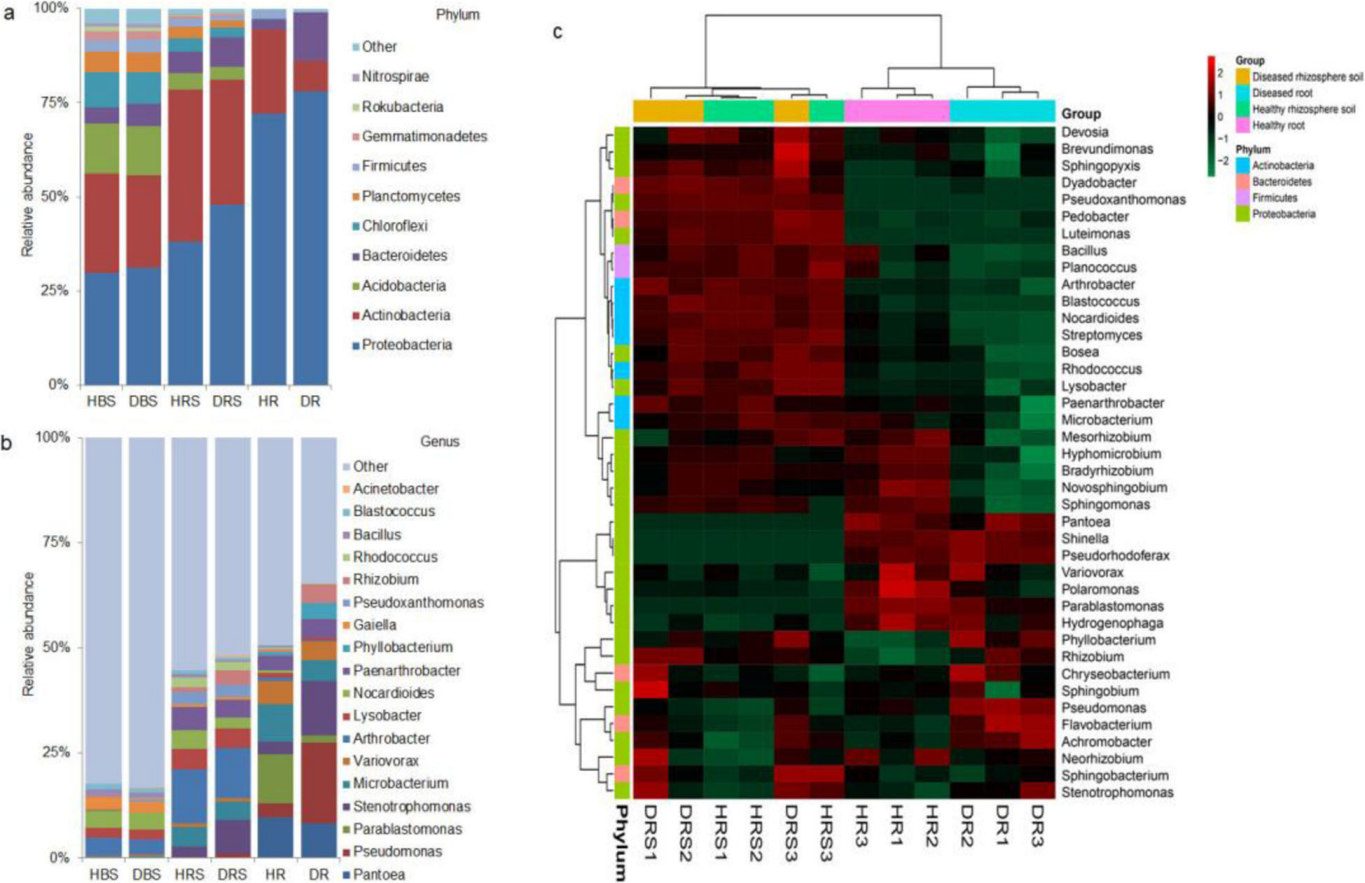
Plant-associated bacterial community composition in different compartments between healthy and diseased plants**. a** Distribution and abundance of major bacterial phyla in different compartments of healthy and diseased plants. **b** Effect of fungal pathogen infection on bacterial community composition and the abundance of major bacterial phyla in different compartments. **c** Heat map of the bacterial community composition with cluster analysis. Similar samples were clustered horizontally, and vertical patterns illustrate the phylogenetic relationships among the top 40 bacterial genera across samples. *HBS*, healthy bulk soil; *HRS*, healthy rhizosphere; *HR*, healthy root; *DBS*, diseased bulk soil; *DRS*, diseased rhizosphere; *DR*, diseased root

**Fig. 4:**
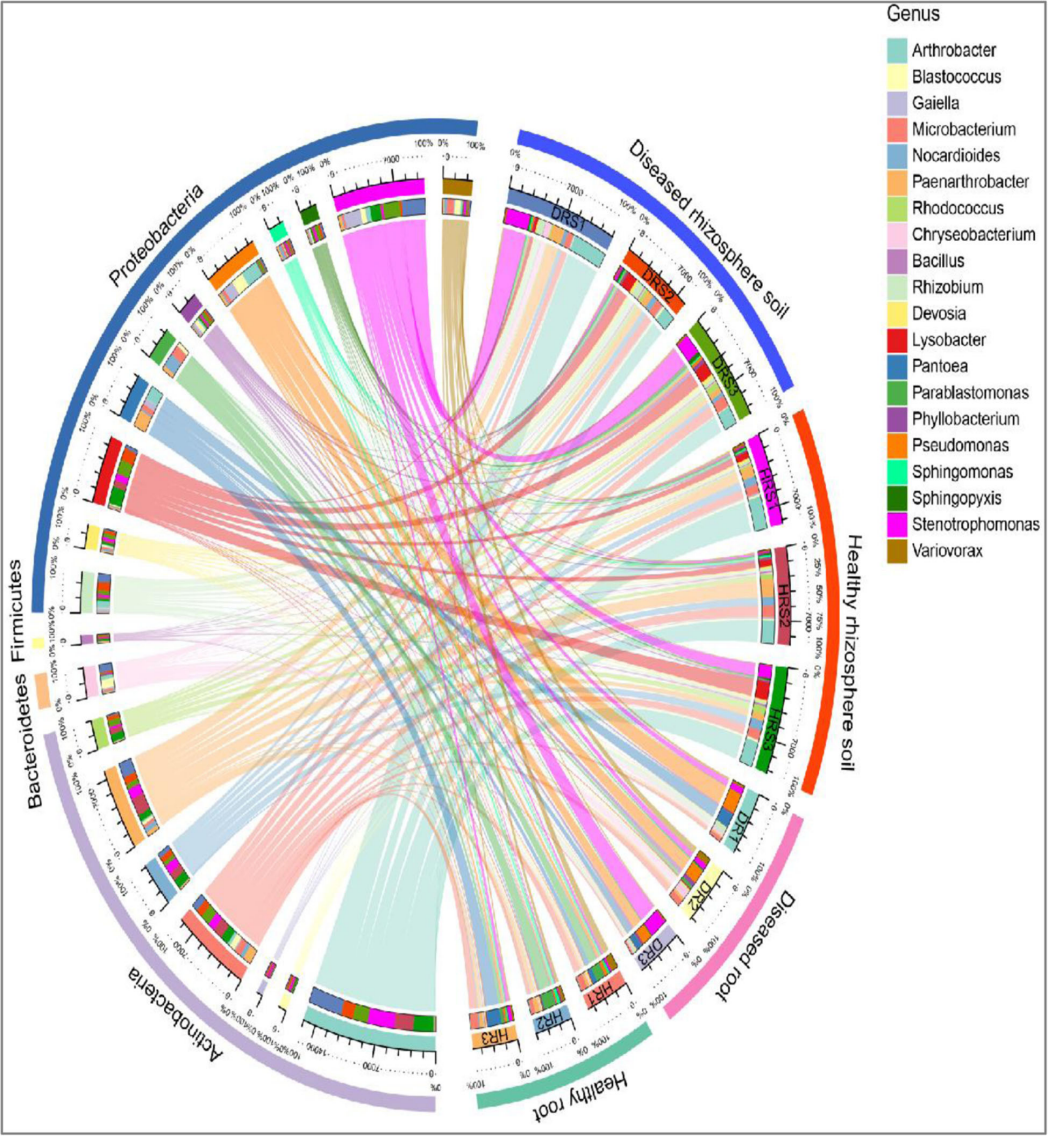
Effects of fungal pathogens on bacterial composition in healthy and diseased plants. The dominant bacterial phyla and genera in diseased and healthy roots and rhizosphere. The left half circle from the outside to the inside represents the dominant phyla, dominant genera, and proportions of each genus in different samples. The right half circle represents the different samples

Based on the OTU classification results, Proteobacteria, Actinobacteria, and Bacteroidetes were the dominant phyla in the roots and rhizosphere, accounting for 86.7–98% of the total reads, while Proteobacteria (30.4%), Actinobacteria (25.6%), Acidobacteria (13.1%), and Chloroflexi (8.8%) dominated the bulk soil bacterial communities (Fig. 3a). Compared with the bulk soil, the rhizosphere was significantly enriched in Proteobacteria and Actinobacteria, while the roots were obviously enriched in Proteobacteria (Fig. s4). *Fusarium oxysporum* infection could also affect the root-associated bacterial community composition. The bacterial phyla that were significantly affected by pathogen infection were Proteobacteria and Bacteroidetes in the roots, and Actinobacteria and Bacteroidetes in the rhizosphere. However, bacterial phylum abundance in bulk soil was not affected by *F. oxysporum* (Fig. 4 and Fig. s5).

At the genus level, the most abundant bacterial genera in bulk soil were *Nocardioides* (3.9%), *Arthrobacter* (3.8%), and *Gaiella* (2.8%). Conversely, *Arthrobacter* and *Stenotrophomonas* were the dominant genera in the rhizosphere, while *Pseudomonas*, *Stenotrophomonas*, and *Pantoea* were the most dominant genera in the roots (Fig. 3b).

**Fig. 5:**
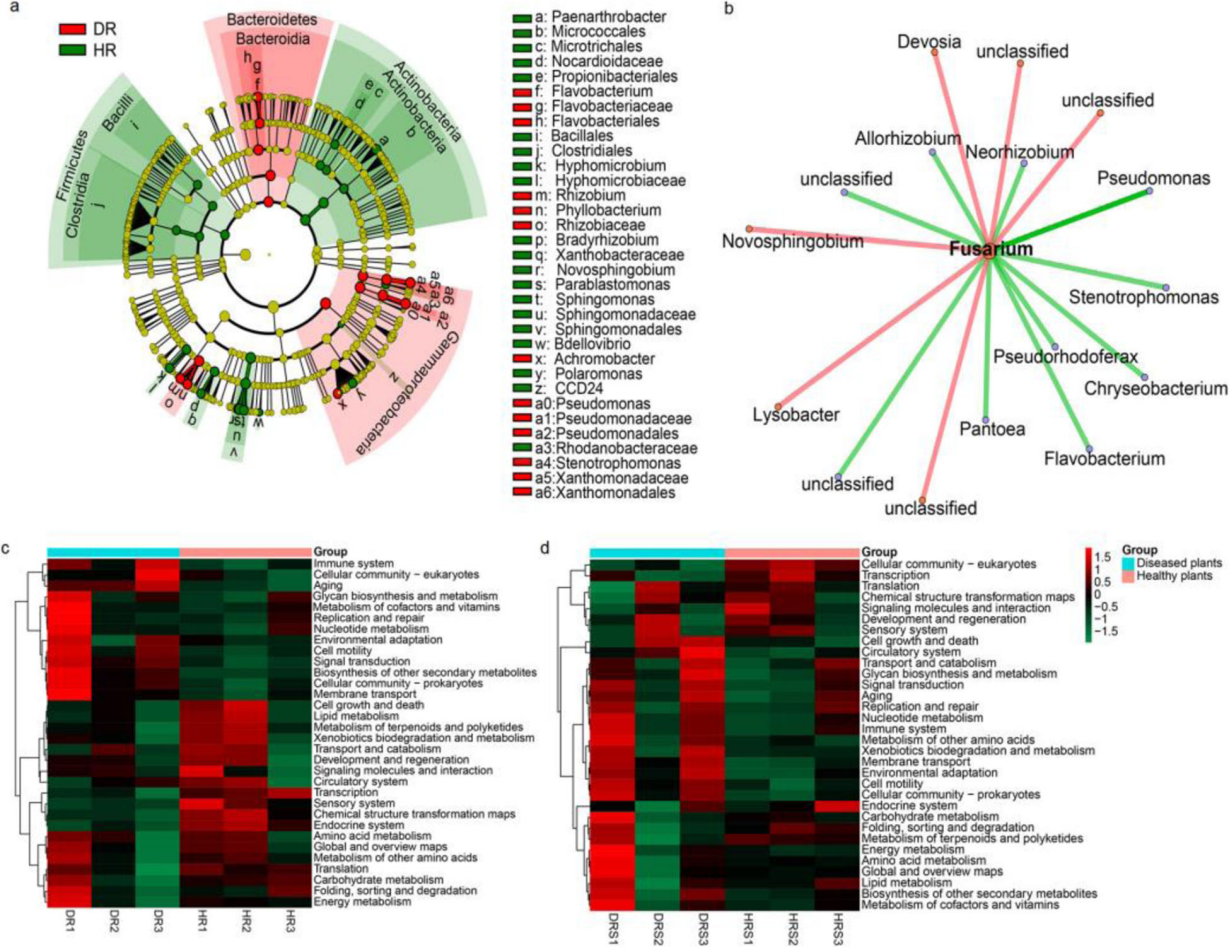
Taxonomic and functional characteristics of bacterial communities after pathogen infection**.** Least discriminant analysis (LDA) effect size taxonomic cladogram comparing bacteria from healthy and diseased samples. The different classification levels are presented from the inside to the outside. The red nodes indicate enriched genera in diseased roots, yellow nodes indicate no difference, and green nodes indicate enriched genera in healthy roots. *HR*, healthy roots; *DR*, diseased roots. **b** The relationship between root-associated bacteria (relative abundance > 0.001) and *Fusarium* was calculated using Spearman correlations. Bacteria with a correlation coefficient greater than 0.6 were used to construct the network. Red and green lines indicate that the relative abundance of the bacteria was positively or negatively correlated with *Fusarium*. **c** The root and **d** rhizosphere functional profiles. The color scale represents enrichment or reduction of the predicted function

The heat map of the top 40 genera revealed that the bacterial communities in the healthy and diseased roots were clustered separately. However, bacterial communities in the rhizosphere were not distinguishable between the healthy and diseased plants (Fig. 3c), indicating that infection with *F. oxysporum* greatly impacted the bacterial community structure in roots more than it did that of rhizospheres. To investigate which bacteria were recruited by plant roots during fungal pathogen infection, we analyzed the distinct bacteria between the healthy and diseased plants. The results showed that *Stenotrophomonas*, *Pseudomonas*, and *Flavobacterium* were highly enriched in diseased roots (Fig. 5a), while *Rhizobiaceae* and *Flavobacteriales* were enriched in diseased rhizospheres (Fig. s6).

### The functional traits of the bacterial communities in diseased rhizospheres and roots

The 16S rRNA sequencing results showed that the bacterial community composition of diseased roots was significantly different from that of healthy roots. To investigate whether bacteria recruited from diseased plants alter the functional profiles of bacterial communities, we first predicted the community functions via PICRUSt software. The bacterial community functional profiles were different between healthy and diseased rhizospheres and roots. In diseased rhizospheres and roots, the functions were related to “environmental adaption”, “cell motility”, “signal transduction,” and “biosynthesis of secondary metabolites” (Fig. 5c and d and Table s3). Next, we analyzed the relationship between the enriched OTUs in diseased roots and fungal pathogens. Interacting network analysis results indicated that *Stenotrophomonas, Flavobacterium*, and *Pseudomonas* abundances were negatively correlated with *Fusarium* growth (Fig. 5b).

### Construction of synthetic communities and determination of disease resistance

To further evaluate the effects of the recruited bacteria on *Astragalus* health, we isolated 423 bacterial isolates from the rhizosphere and roots of healthy and diseased *Astragalus* using 0.1 ×, 0.2 ×, and 0.5 × TSA, R2A and LB plates. The isolates were from 74 genera, according to the 16S rRNA sequencing results. Among them, 44 isolates could solubilize organic P, 65 isolates could solubilize inorganic P, 40 isolates could solubilize K, and 80 isolates could produce IAA (Fig. s7).

**Fig. 6:**
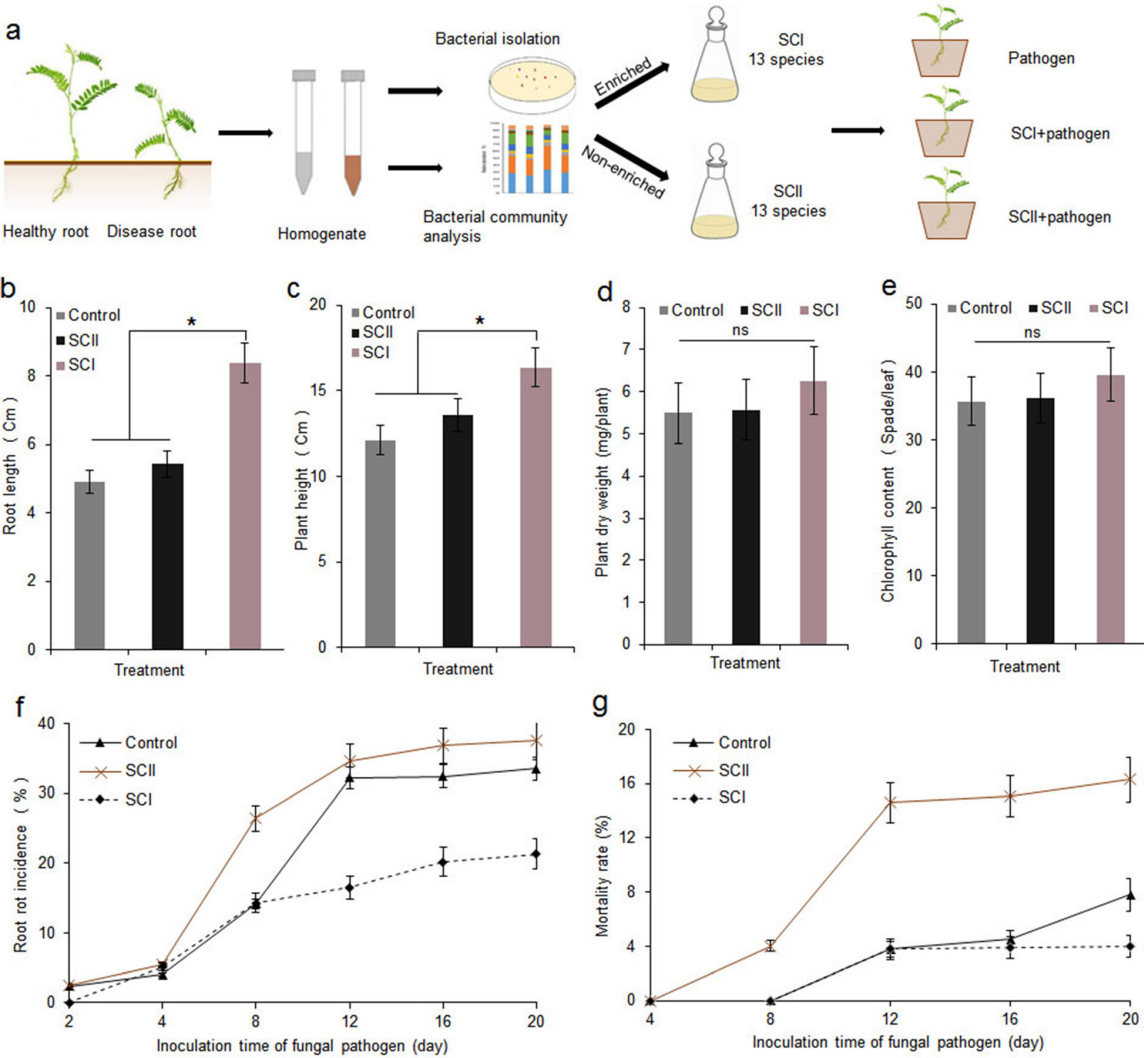
Synthetic bacterial community assembly and the regulatory effects of synthetic communities on *Astragalus* root rot. **a** Processes of establishing synthetic bacterial communities. Bacteria were isolated from the rhizosphere and roots by culturable methods as much as possible, and the different strains of healthy and diseased roots were analyzed. Subsequently, the synthetic community I (SCI) and synthetic community II (SCII) were assembled with enriched and non-enriched bacteria in diseased roots, respectively. **b**–**e** Effects of SCI and SCII on root length, plant height, chlorophyll content (*n* = 20), and plant dry weight (*n* = 4) 20 days after inoculation. The synthetic bacterial communities were inoculated 5 days before the fungal pathogens were introduced into the soil, and each plant was inoculated with a 2-mL suspension of synthetic communities (~ 10^8^ CFU/mL). Asterisks indicate significant differences (*P* < 0.05). **f**–**g** Effects of SCI and SCII on root rot incidence and plant mortality at different time points after inoculation (*n* = 45). Each plant was inoculated with a 2-mL spore suspension of *F. oxysporum* (~ 10^5^ spores/L)

With a relative abundance ratio of 2 as the threshold, 39 bacterial genera were enriched, and 137 bacterial genera were depleted in diseased roots (Tables s4 and s5). *F. oxysporum* infection led to a significant increase in bacterial genera such as *Pseudomonas*, *Strenotrophomonas*, *Chryseobacterium*, *Achromobacter*, and *Flavobacterium* in roots and a decrease in *Pantoea*, *Parablastomonas*, and *Microbacterium* (Fig. 5a). We isolated 16 out of the 39 enriched genera and 11 out of the 137 depleted genera, and each genus contained two to eight species. We then selected one species from 10 high-abundance (> 0.1%) and three low-abundance genera (< 0.1%) based on their antagonistic or plant growth–promoting characteristics. We then mixed all the species with equal-volume suspensions (~ 10^8^ cells/L) and generated synthetic community I (SCI, Fig. s8 and Table s1). To investigate whether non-enriched bacteria could prevent fungal pathogens from infecting *Astragalus*, we also selected nine species with decreased relative abundance and four random species to construct synthetic community II (SCII) (Fig. 6a, Fig. s9, and Table s1).

Plants treated with the two synthetic communities grew for 20 days, and the root lengths in SCI-treated plants were 70.50% and 54.33% greater than the lengths of the uninoculated and SCII-treated plants, respectively, while the plant heights of SCI-treated plants were 34.48% and 20.56% greater than the heights in the control and SCII-treated plants, respectively (Fig. 6b and c). There were no significant differences in root length or plant height between the SCII-treated and control plants. In addition, neither SCI nor SCII treatment influenced plant fresh weight, dry weight, and chlorophyll contents (Fig. 6d and e).

SCI and SCII also had different effects on root rot incidence (Fig. 6f and g), based on the results of the pot experiments. After 12 days of inoculation with *F. oxysporum*, SCI significantly reduced the root rot incidence in *Astragalus*. The average incidence and mortality of SCI-treated plants were 26.32% and 23.64% lower than those of the control at 20 days after pathogen inoculation.

### Simplification of the complex bacterial community

**Fig. 7:**
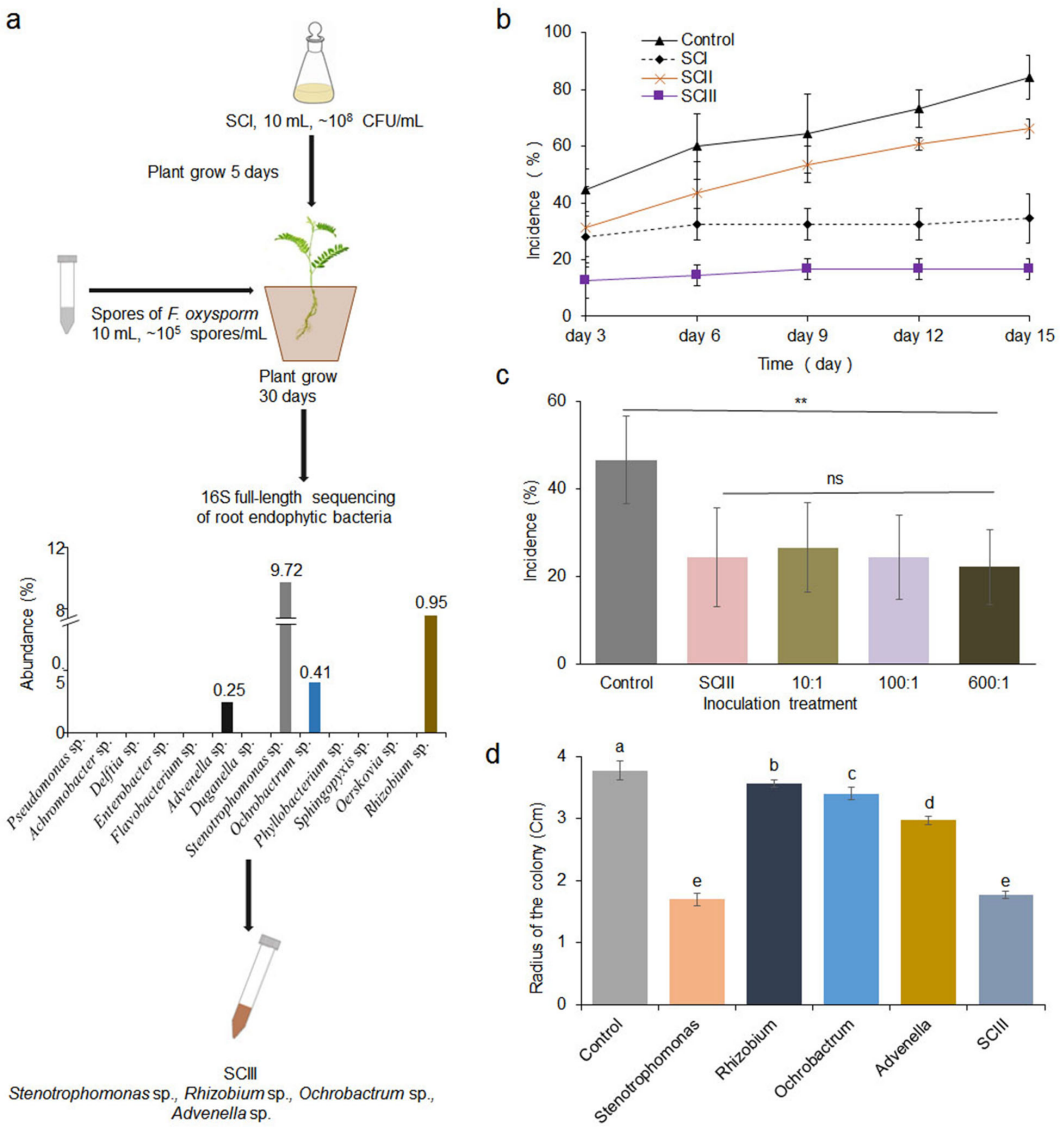
Simplification of synthetic bacterial community I and disease control effects**. a** SCIII was constructed by mixing *Stenotrophomonas* sp., *Rhizobium* sp., *Advenella* sp., and *Ochrobactrum* sp. that survived joint selection by the host plant and *F. oxysporum*. The bar graph shows the abundance of the four bacterial strains in roots. **b** Root rot disease incidence in plants inoculated with different bacterial communities for 15 days. *SCI*, synthetic community I; *SCII*, synthetic community II; *SCIII*, four-species simple community. **c** Colony radii of *F. oxysporum* treated with different bacteria after 6 days of growth (*n* = 3). **d** Root rot incidence of *Astragalus* inoculated with mixed bacteria at different ratios. Asterisks indicate significant differences (Tukey test, *P* < 0.01). 10:1, *Stenotrophomonas*:*Rhizobium*:*Ochrobactrum*:*Advenella* = 10:10:1:1; 100:1, *Stenotrophomonas*:*Rhizobium*:*Ochrobactrum*:*Advenella* = 100:100:1:1; 600:1, *Stenotrophomonas*:*Rhizobium*:*Ochrobactrum*:*Advenella* = 600:200:10:1

After SCI was inoculated in soil for 30 days, only *Stenotrophomonas* sp., *Rhizobium* sp., *Advenella* sp., and *Ochrobactrum* sp. could be recovered from the roots and rhizospheres, and *Stenotrophomonas* sp. was the dominant species (Fig. 7a). However, it is interesting that the simple community (SCIII) assembled with the four species could also decrease root rot incidence and plant mortality (Fig. 7b, *n* = 12, *P* < 0.001, Tukey test). Furthermore, the numbers of diseased seedlings treated with SCI and SCIII were significantly lower than those in the untreated control and SCII-treated plants. No obvious differences in the number of diseased seedlings were found between the SCI- and SCIII-treated plants (Fig. 7b and Fig. s10). We also tested the control effects of the mixture of high-abundance bacteria and low-abundance bacteria at different proportions when the simple synthetic community was established. The results showed that reducing the amount of *Advenella* sp. and *Ochrobactrum* sp. did not affect the regulatory effects of simple synthetic communities on *Astragalus* root rot (Fig. 7c).

### Fungal pathogen growth inhibition and plant disease control by the four bacteria

*Stenotrophomonas* sp. could directly inhibit the growth of fungal mycelia; however, *Advenella* sp., *Rhizobium* sp., and *Ochrobactrum* sp. had a minimal negative effect on *F. oxysporum* growth, which was consistent with interacting network analysis showing that *Stenotrophomonas*, *Flavobacterium*, and *Pseudomonas* abundance was negatively correlated with *Fusarium* growth (Figs. 5b and 7d). Although the colony radii of fungal pathogens treated with *Advenella* sp., *Rhizobium* sp., and *Ochrobactrum* sp. were smaller than those of the control fungi after 6 days of growth, no inhibitory effect was observed on Day 10 (Fig. s10). Interestingly, SCIII and *Stenotrophomonas* sp. alone had similar inhibitory efficiency against *F. oxysporum*, indicating that the inhibitory effect of SCIII on fungal growth did not increase with the addition of three bacterial strains (*Advenella* sp., *Rhizobium* sp., and *Ochrobactrum* sp.) in PDA agar plates. Therefore, we speculated that *Stenotrophomonas* sp. protected *Astragalus* by inhibiting *F. oxysporum* growth, while the other three strains prevented the fungal pathogens from invading *Astragalus* by different means, instead of inhibiting fungal mycelial growth in plants.

**Fig. 8:**
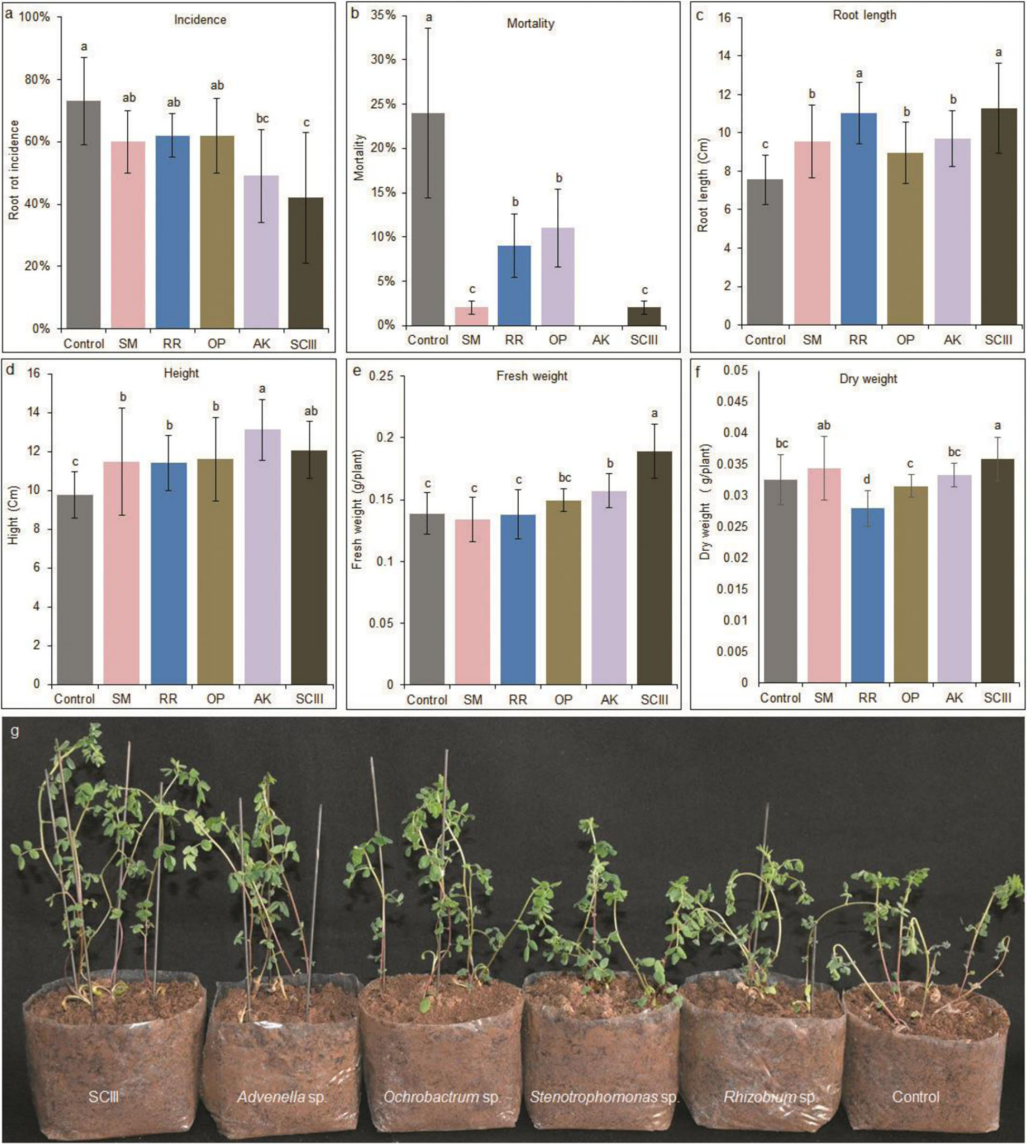
Effect of SCIII and single bacterium inoculation on plant growth, root rot incidence, and mortality**. a** Root rot disease incidence and **b** mortality of plants inoculated with a single bacterium or SCIII after 10 days of inoculation (*n* = 45). Each plant was inoculated with 2 mL of bacterial suspension (~ 10^8^ CFU/mL). After 5 days, each plant was inoculated with a 2-mL spore suspension of *F. oxysporum* (~ 10^5^ spores/L). **c**–**f** Effects of different single-bacterium groups and SCIII on root length, height, fresh weight, and dry weight of *Astragalus*. *SM*, *Stenotrophomonas* sp.; *RR*, *Rhizobium* sp.; *OP*, *Ochrobactrum* sp.; *AK*, *Advenella* sp.; *SCIII*, four-species synthetic community. Letters indicate significant differences (n = 20, *P* < 0.05). Each plant was inoculated with 2-mL bacterial suspensions and grown for 15 days. **g** Phenotypes of *Astragalus* inoculated with single bacteria or SCIII after 15 days of growth in a greenhouse

It seems that *Stenotrophomonas* sp. is the only bacterial species from SCIII that can inhibit the growth of *F. oxysporum*. We were curious to know if the presence of the three other bacterial species, *Advenella* sp., *Rhizobium* sp., and *Ochrobactrum* sp., plays a role in the control of root rot disease. Therefore, we examined the effects of root rot incidence in *Astragalus* by using single species in comparison to SCIII. Although all four species led to an obvious reduction in plant mortality, *Stenotrophomonas* sp., *Rhizobium* sp., and *Ochrobactrum* sp. did not significantly decrease the root rot disease incidence in host plants (Fig. 8a and b). Only *Advenella* sp., with the lowest abundance of all four species, significantly reduced plant disease incidence (~ 33.3%) and mortality (~ 100%). However, the effects of root rot incidence of SCIII with a decrease of 42.7% were superior to those of any other single bacteria, suggesting that the synthetic community worked better than single species in defending against pathogen infection. Moreover, all inoculation treatments could promote plant growth. *Rhizobium* sp., *Advenella* sp., and SCIII increased plant height and root length the most (Fig. 8c and d); in addition, SCIII could significantly improve plant fresh and dry weights (Fig. 8e and f).

### Induction of systemic resistance against *Astragalus* by synthetic communities

We noticed that single species, SCI, and SCIII could significantly increase endogenous JA contents in plants. Although JA contents fluctuated in each treatment at different times, they remained much higher in the plants treated with either *Advenella* sp. or *Ochrobactrum* sp. than in the plants treated with *Stenotrophomonas* sp. or *Rhizobium* sp. SCIII induced more JA than any of the single species (Fig. s11 and Table s6).

We also examined the activities of PAL, PPO, and chitinase after inoculating the plants with synthetic communities or single species. All treatments gradually increased the activities of PAL, PPO, and chitinase and peaked at 168 h after inoculation. The activities of PAL, PPO, and chitinase in plants inoculated with *Advenella* sp. and *Ochrobactrum* sp. were higher than those of plants inoculated with *Stenotrophomonas* sp.. and *Rhizobium* sp. could also induce higher PAL activity only after 72 and 168 h and higher PPO activity than nontreated plants after 24 h. SCIII was found to increase the PAL, PPO, and chitinase activities when compared with the nontreated control by 31.9%, 40.8%, and 21.4%, respectively (Fig. s11b-s11d and Table s6).

SCI, SCIII, and single species can also rapidly increase POD and LOX enzyme activities after 8 h post inoculation, followed by a decrease. At 168 h post inoculation, the activities of POD and LOX reached their maximum. *Advenella* sp. and SCIII could significantly increase the activities of POD and LOX in the plants when compared to other single species and nontreated controls. At 168 h post inoculation, the activities of POD in *Advenella* sp. and SCIII-treated plants increased by 22.5% and 29.4%, respectively, and the activities of LOX increased by 46.1% and 39.7%, respectively (Fig. s11e–s11f, Table s6).

**Fig. 9:**
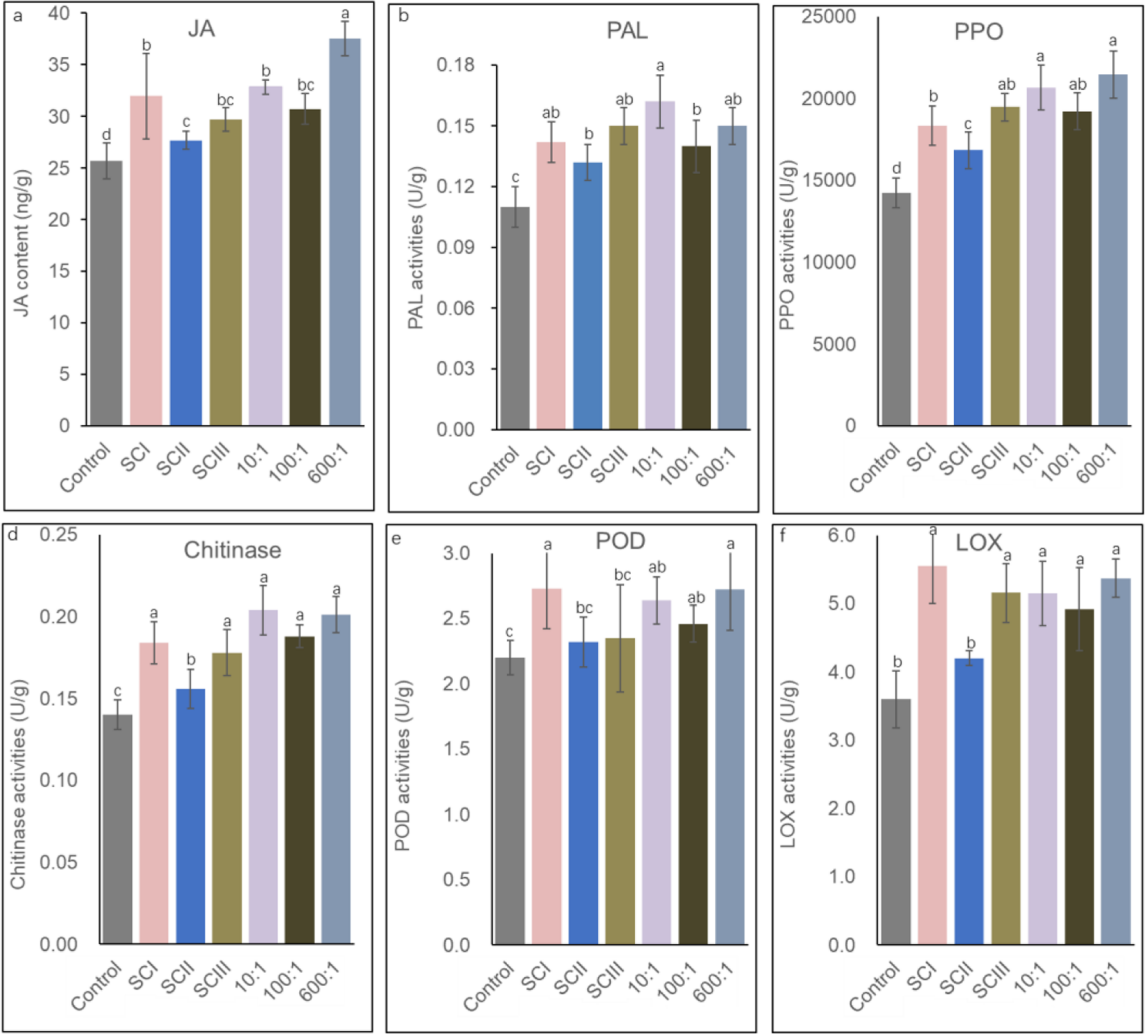
Effects of different synthetic communities on the ISR of Astragalus. JA contents and PAL, POD, PPO, LOX, and chitinase activities of plants on the 5th day after inoculation with sterile water, SCI, SCII, SCIII, and different mixing ratios of four bacterial species. The letters above the bars denote differences based on Duncan’s multiple range test (*n* = 3, *P* < 0.05). *JA*, jasmonic acid; *PAL*, phenylalanine ammonia lyase; *PPO*, polyphenol oxidase; *POD*, peroxidase; *LOX*, lipoxygenase; *ISR*, induced systemic resistance; 10:1, *Stenotrophomonas*:*Rhizobium*:*Ochrobactrum*:*Advenella* = 10:10:1:1; 100:1, *Stenotrophomonas*:*Rhizobium*:*Ochrobactrum*:*Advenella* = 100:100:1:1; 600:1, *Stenotrophomonas*:*Rhizobium*:*Ochrobactrum*:*Advenella* = 600:200:10:1

We then compared the activation of plant ISR by SCI, SCII, SCIII, SCIII-10, SCIII-100, and SCIII-600 (high- and low-abundance bacteria mixed at ratios of 10:1, 100:1, and 600:1). Five days post inoculation, no significant differences in POD, PPO, PAL, LOX, and chitinase activities were found among the plants treated with SCI, SCIII, and SCIII with different mixing ratios. Although all the enzyme activities of SCII-treated plants were higher than those of the nontreated control, they were obviously lower than those in other synthetic community-treated plants (Fig. 9).

## Discussion

### Recruitment of beneficial bacteria by host plants under fungal pathogen infection

Environmental conditions such as drought, fertility, pH, pathogenic fungi, and nematodes can alter the metabolic pathways of plants and induce the production of specific root exudates, which selectively recruit microorganisms from the surrounding soil to colonize the rhizosphere or roots [48, 49]. The resulting complex microbial communities could enhance the host plant’s resistance or tolerance to adverse environments. The composition and assembly of the microbial community in the rhizosphere of wheat, maize, paddy, sorghum, and other plants under biotic and abiotic stress have been extensively studied [50–52]. However, it remains unclear which members of the microbial communities directly influence plant performance. A potential approach for determining the key microbes influencing plant performance under such conditions is to simulate natural conditions, establish synthetic microbiota, and analyze their correlation with host plant growth, metabolism, and stress tolerance.

In the present study, we found that bacterial community alpha diversity in roots was significantly lower than that in the rhizosphere and bulk soil regardless of the presence or absence of *F. oxysporum*, which is consistent with the findings in a previous study showing that plant root systems had a strong filtration effect on soil microorganisms [53]. Pathogenic fungal infection significantly decreased the Shannon diversity of root endophytic bacterial communities. This could be due to the high enrichment of bacteria associated with disease defense in the roots. Compared with the bacterial communities in healthy plants, *Pseudomonas*, *Stenotrophomonas*, *Arthrobacter*, and *Flavobacterium* were highly enriched in the roots of diseased *Astragalus* plants. *Pseudomonas* and *Stenotrophomonas* were the most enriched species, with their abundance increasing from 3.33% and 2.97% in healthy roots to 19.17% and 13.19% in diseased roots, respectively. The top 10 enriched bacterial genera in diseased roots accounted for ~ 59.6% of the root endophytic bacterial communities. Many strains in these genera have been reported to promote plant growth and improve plant resistance to disease [54, 55]. The functional profiles of the root-associated bacterial communities showed different patterns among the samples. We found that some functions of root-associated communities were related to the pathogen resistance of the plant, including “biofilm formation”, “streptomycin biosynthesis”, “polyketide biosynthesis”, and “lipopolysaccharide biosynthesis”. Biofilm can help plants absorb nutrients and act as biocontrol agents to fight against diseases. Downy mildew infection in *Arabidopsis* leads to the formation of a three-species bacterial consortium that interacts synergistically in biofilm formation and decreases disease incidence [16]. Polyketide and streptomycin are commonly used antibiotics for inhibiting the growth of bacteria and fungi, and the expression of polyketide cyclase genes is significantly increased under pathogen stress [56]. Lipopolysaccharides produced by gram-negative bacteria can act as elicitors that can induce defense responses in plants [57]. The results indicate that *F. oxysporum* infection could alter the assembly of root-associated microbial communities and recruit specific microorganisms that control root rot disease in *Astragalus*. In our study, most of the bacteria were isolated from both diseased roots and healthy roots, indicating that the communities assembled on the roots of healthy plants also have the potential to control the disease. However, plants are exposed to stress from various biotic and abiotic factors simultaneously when they grow in natural conditions. In the present study, microbes with different ecological functions were enriched in the rhizospheres and roots. Even under pathogen inoculation, the microorganisms enriched in plants were not all associated with disease resistance. The combination of different bacteria and determination of the compatible and ecologically stable communities are laborious and time-consuming tasks. By analyzing the microbial community composition of diseased plants, we can select microbial strains for establishing appropriate combinations, which could improve the efficiency of assembling disease-resistant microbial communities.

### Construction and simplification of synthetic bacterial communities

Microbial diversity in soil is much greater than that observed from 16S high-throughput sequencing. It remains unclear how many types of microbes can be recruited by plants from the soil to perform specific functions. In early studies, the number of bacteria in synthetic communities can range from several to dozens [21, 58]. It is also not clear whether some or all the recruited bacteria are involved in plant disease resistance. Therefore, the construction of synthetic bacterial communities could help answer the questions posed above.

In the present study, we found that an artificial synthetic bacterial community (SCI) composed of 13 out of 39 bacterial genera significantly enriched in diseased *Astragalus* roots could considerably alleviate plant disease. Further investigations revealed that the control effect of the synthetic community on plant root rot remained with only four bacterial species from SCI, suggesting that not all the enriched bacteria participated in the resistance to fungal pathogens. In our study, we found that *Stenotrophomonas* sp., *Rhizobium* sp., *Advenella* sp., and *Ochrobactrum* sp. were the key species in the control of plant root rot disease. The relative abundance ratio of *Stenotrophomonas* sp., *Rhizobium* sp., *Ochrobactrum* sp., and *Advenella* sp. was approximately 600:200:10:1. We also tested different ratios of the four species on root rot and found that this four-species community was effective in preventing root rot despite variable proportions. This result indicated that some bacteria could also play a role at low concentrations. To attract associated microbes in the soil, plants need to excrete 5–21% of photosynthetic products via their roots [59, 60]. In addition, the more microorganisms are enriched in the rhizosphere or roots, the greater resources are required to maintain the growth and reproduction of the microorganisms; thus, it is resource intensive for plants to host numerous microbes. In addition, although the colonization of the roots or the rhizosphere by microbial communities is an essential step for the control of soil-borne disease [61], the presence of numerous strains in a community could increase the competition among them. Therefore, plants tend to enrich a few microorganisms to perform specific functions. For example, *Arabidopsis* with downy mildew recruits *Microbacterium* sp., *Stenotrophomonas* sp., and *Xanthomonas* sp. in the rhizosphere [16], and Niu et al. observed that a seven-strain bacterial community delayed the growth of *Fusarium verticillioides* [58]. Therefore, the optimal strain combination rather than more strains should be emphasized when constructing a synthetic community.

Several species were obtained within a genus in the process of isolating the plant-recruited bacteria through culturable methods; consequently, it is essential to select the appropriate bacterial species for synthetic community assembly. Considering that beneficial microorganisms can improve plant disease resistance through growth promotion and antagonism, we selected bacteria with such characteristics for use in the assembly of bacterial communities. SCI was remarkably effective in the control of root rot disease. However, SCII did not alleviate root rot disease in *Astragalus* even though it contains *Bacillus velezensis* and *Paenibacillus terrae*, species that can inhibit the growth of pathogenic fungi by producing bioactive substances [62, 63]. Although SCI could decrease the incidence of *Astragalus* root rot disease, we found that the synthetic community containing four species had the same effect on root rot disease control. Thus, whether the other bacterial species from SCI could participate in the alleviation of root rot disease needs to be verified. Today, numerous biocontrol agents are prepared on the premise that the candidate strains are antagonistic against pathogens in the laboratory; however, such effects may not be observed in the field. Therefore, the study of the assembly of synthetic bacterial communities based on bacteria recruited by the plant facilitates the stress resistance of the host plant [16, 64].

### Low- and high-abundance bacteria in synthetic communities jointly enhance plant disease resistance

Plants recruit diverse microbes to control plant disease via antibiotic production, competition for nutrients, plant growth promotion, or systemic resistance induction [65–68]. Some studies have reported that a decrease in rhizosphere microbial community diversity and the abundance of some species made the plants more susceptible to pathogen infection [69, 70]. However, the influence of the relative abundances of the components in synthetic communities cannot be excluded [21, 57]. The existence of low-abundance microorganisms also helps to accommodate more types of microbes with limited root exudates, which may be beneficial for plant growth, stress tolerance, and overall plant health [71]. In addition to their roles in inhibiting fungal pathogens and promoting plant growth, bacterial communities can also activate the immunity of plants to prevent pathogen infections. Therefore, we have also studied the effects of different synthetic communities on the ISR of *Astragalus*. Except for SCII, SCI and SCIII can significantly activate the ISR of plants and reducing the abundance of *Ochrobactrum* sp. and *Advenella* sp. did not weaken the control effect of SCIII. Although the low abundance of *Ochrobactrum* sp. and *Advenella* sp. could not directly inhibit *F. oxysporum* growth, it conferred a greater activation effect on the JA and ISR-related enzyme activities in plants than *Stenotrophomonas* sp., suggesting that these two low-abundance bacteria may induce plant resistance via the JA signaling pathway. Plants lacking the JA synthesis metabolic pathway are more susceptible to pathogen infection, while the incidence rates of infection can be significantly reduced by increasing JA accumulation [72].

It has been reported that some beneficial rhizosphere bacteria can activate the JA synthesis pathway during interactions with plants [73]. JA-induced pathways also produce chitinases and oxidative enzymes (for example, PAL, POD, PPO, and LOX). Increased PAL and POD activities could enhance the production of phenolics and lignification to prevent pathogen invasion [74–76].

Overall, our findings suggest that high-abundance bacteria could protect hosts by promoting plant growth and inhibiting pathogenic fungal growth, while low-abundance bacteria control diseases by enhancing plant ISR. Moreover, the combination of both high- and low-abundance bacterial species induced greater systemic resistance than any single species. Our findings could provide a reference to facilitate the assembly of functional synthetic communities and highlight a potential solution that could be applied to achieve sustainable agriculture.

## Conclusions

Using next-generation sequencing methods, we showed that the composition of the root endophytic bacterial community of diseased plants was distinctly different from that of healthy plants. Particularly, under *F. oxysporum* challenge, the plant roots recruit some beneficial microbes, such as *Pseudomonas*, *Strenotrophomonas*, *Chryseobacterium*, *Achromobacter*, and *Flavobacterium*. The assembly of three low-abundance and 10 high-abundance bacteria enriched in diseased roots can effectively control *Astragalus* root rot disease as a synthetic community. A simplified synthetic community based on the above community but containing only four bacteria maintained the original effect. Further investigation showed that the high-abundance bacteria were responsible for inhibiting the growth of fungal pathogens, while the low-abundance bacteria were mainly associated with activating plant ISR to alleviate *Astragalus* root rot disease. The findings suggest that bacteria with different ecological functions can be assembled as synthetic bacterial communities for soil-borne disease control. Such functional synthetic communities can be used as a possible solution for sustainable agriculture.

## Supplementary Information

**Additional file 1: **Supplementary materials. **Fig. S1.** Schematic diagram of root and soil sample collection. **Fig. S2.** The distinct and shared bacterial genera in different sample compartments. **Fig. S3.** Effect of fungal pathogen on Shannon diversity of bacterial communities in bulk soil, rhizosphere and root. **Fig. S4.** Enrichment of bacterial phyla by *Astragalus* roots. **Fig. S5.** Effect of *F. oxysporum* infection on the abundance of major bacterial phyla. **Fig. S6.** Least discriminant analysis (LDA) effect size taxonomic cladogram comparing healthy and diseased rhizosphere samples. **Fig. S7.** Plant growth-promoting properties of plant-associated bacteria. **Fig. S8.** Relative abundance of bacteria in SCI found in healthy and diseased roots. **Fig. S9.** Relative abundance of bacteria in SCII found in healthy and diseased roots. **Fig. S10.** Inhibition of four bacteria in simple synthetic community (SCIII) on the growth of *F. oxysporum*. **Fig. S11.** Effect of SCIII and single bacterium inoculation on the ISR of *Astragalus*.

**Additional file 2: Table S1.** Characteristics of bacteria in two synthetic communities.

**Additional file 3: Table S2**. Diversity index of bacterial community in different compartment of *A. mongholicus*.

**Additional file 4: Table S3.** Root and rhizosphere community KEGG pathways.

**Additional file 5: Table S4.** Abundance of bacterial genera increased in diseased roots.

**Additional file 6: Table S5.** Abundance of bacterial genera decreased in diseased roots.

**Additional file 7: Table S6.** Effects of different inoculation and treatment time on Astragalus JA content and ISR-related enzyme activities.

## Data Availability

Raw data used in this study are available in the NCBI Sequence Read Archive (SRA), accession numbers PRJNA762455 and PRJNA764034.

## Abbreviations

JA: Jasmonic acid
POD: Peroxidase
PPO: Polyphenol oxidase
PAL: Phenylalanine ammonia lyase
LOX: Lipoxygenase
IAA: Indole-3-acetic acid
PDA: Potato dextrose agar
ISR: Induced systemic resistance
Ir: Inhibition ratio
SCI: Synthetic community I
SCII: Synthetic community II
SCIII: Four-species community
