# Supplementary material for: A simplified synthetic community rescues Astragalus mongholicus from root rot disease by activating plant-induced systemic resistance
Source: Microbiome. 2021 Nov 4;9:217. doi: 10.1186/s40168-021-01169-9 (PMC8567675; doi:10.1186/s40168-021-01169-9)
Supplement: Supplementary file 2 — Additional file 1: Supplementary materials. Fig. S1. Schematic diagram of root and soil sample collection. Fig. S2. The distinct and shared bacterial genera in different sample compartments. Fig. S3. Effect of fungal pathogen on Shannon diversity of bacterial communities in bulk soil, rhizosphere and root. Fig. S4. Enrichment of bacterial phyla by Astragalus roots. Fig. S5. Effect of F. oxysporum infection on the abundance of major bacterial phyla. Fig. S6. Least discriminant analysis (LDA) effect size taxonomic cladogram comparing healthy and diseased rhizosphere samples. Fig. S7. Plant growth-promoting properties of plant-associated bacteria. Fig. S8. Relative abundance of bacteria in SCI found in healthy and diseased roots. Fig. S9. Relative abundance of bacteria in SCII found in healthy and diseased roots. Fig. S10. Inhibition of four bacteria in simple synthetic community (SCIII) on the growth of F. oxysporum. Fig. S11. Effect of SCIII and single bacterium inoculation on the ISR of Astragalus. [file 40168_2021_1169_MOESM2_ESM.docx]

**A simplified synthetic community rescues *Astragalus mongholicus* from root-rot disease by activating plant-induced systemic resistance**

Zhefei Li^1, 2, #, *^, Xiaoli Bai^1, #^, Shuo Jiao^1, 2^, Yanmei Li^1^, Peirong Li^1^, Yan Yang^1^, Hui Zhang^1^, Gehong Wei^1, 2, *^

^1^State Key Laboratory of Crop Stress Biology in Arid Areas, Northwest A&F University, Yangling, Shaanxi 712100, China

^2^Shaanxi Key Laboratory of Agricultural and Environmental Microbiology, College of Life Science, Northwest A&F University, Yangling, Shaanxi 712100, China

^#^Zhefei Li and Xiaoli Bai contributed equally to this work.

*Correspondent authors:

Zhefei Li: lizhefei@hotmail.com

Gehong Wei: weigehong@nwafu.edu.cn

Tel: +86 29 87081416

Fax: +86 29 87092262

**Materials and methods**

**Isolation of fungal pathogens and plant-associated bacteria**

The field-grown healthy *Astragalus* and the plants displaying symptoms of root rot disease were collected and brought to the laboratory within 24 h of excavation. The roots were shaken violently to remove the loosely bound soil. The roots were placed in a 250 mL conical flask with 50 mL sterile water. The conical flasks were oscillated (180 r / min) at room temperature for 10 min to prepare suspension of rhizosphere microbes. Roots were disinfected with 75% ethanol for 2 min, followed by 3% sodium hypochlorite for 1 min, and rinsed them with sterile water for 6 times. Surface-disinfected roots were ground into homogenate in the autoclaved mortar. Then the endophytic and rhizosphere microbial suspensions of 10^-1^ - 10^-7^ were prepared by 10 - fold dilution. For the bacterial isolation, 100 μL rhizosphere or root suspension of 10^-5^, 10^-6^, 10^-7^ were spread on the surface of 0.1 ×, 0.2 ×, 0.5 × and 0.01 × TSA, R2A and LB plates. For the pathogenic fungal isolation, 100 μL diseased root suspension of 10^-2^ and 10^-3^ were spread on the surface of 1 × PDA plates. All plates were kept in the incubator at 28℃ for 3–7 days, the colonies with different morphological characteristics were picked out and purified by plate streaking. The purified microbial isolations were identified using 16S or 18S rRNA sequence using 27F (AGAGTTTGATCCTGGCTCAG)/1429R (GGTTACCTTGTTACGACTT) or 18sF (AGGCGCGCAAATTACCCAATCC)/18sR (GCCCTCCAATTGTTCCTCGTTAAG), respectively. Then the bacteria and fungal spores were stored in -80 °C freezer.

**Antagonistic activity against pathogenic fungi**

Antagonistic activity of SCIII against pathogenic fungi was determined by evaluating fungal growth inhibition using a confrontation bioassay. A 5-mm mycelial disc of *F. oxysporum* was placed in the center of a potato dextrose agar (PDA) plate and grown in an incubator for 2 d. Five-microliter suspensions of each bacterium in the SCIII were streaked on the surfaces of the media 2.5 cm away from the center of the plate. A similar volume of sterile water was dropped on the PDA plate as the control. All the plates were incubated at 28℃ for 5 d and hypha growth inhibition ratio was calculated using the following equation:

Ir = [(Rw-Rb)/Rw] × 100%

(Rw: the fungal colony radius of the control, Rb: the fungal colony radius of the bacterium).

**Assays of phosphate and potassium solubilization**

Bacteria were inoculated in the center of PVK medium, Mongina organic culture medium with lecithin, and Aleksandrov agar plates [1], respectively. Each treatment had three replicates and all the plates were incubated at 28°C for 7 days. A clear zone around the colony indicated that the bacterium had phosphate or potassium solubilization activity. The solubilization capacity was calculated by Diameter of zone of clearance / Diameter of colony.

**IAA Production**

Isolates were inoculated in 5 mL King B liquid medium supplemented with tryptophan (500 mg/L) and cultivated at 28°C for 48 h on a rotary shaker. Suspension was centrifuged at 12,000 ×*g* for 10 min. The supernatants were mixed with equal volume cold methanol, and the mixtures were incubated at -20°C for 30 min. Then the samples were centrifuged at 12,000 ×g for 15 min. The supernatants were dried and the residues were dissolved with 1 mL methanol. The samples were filtered with 0.45 μL Millipore for HPLC analysis. HPLC method was performed as Sheikhian (2016).

**Induced systemic resistance (ISR) assays**

**Determination of** **jasmonic acid (JA)**

JA was determined as described by Fang et al. (2020) with minor modifications. One gram four-week-old *Astragalus* sample placed into 5 mL 80% methanol and homogenized in the presence of liquid nitrogen. The homogenate was transferred to tubes with stoppers and paced in the sonic bath for ultrasonic extraction for 6 h. All the tubes were kept at 4 °C overnight, and then the mixture were centrifuged at 14,000 ×g for 15 min. The supernatants were evaporated the solvent under vacuum, and the precipitation was dissolved in 1.5 mL 80% methanol. The JA content was measured using plant jasmonic acid ELISA kit according to manufacturer’s protocal (Sinobestbio, Shanghai, China).

**Determination of phenylalanine ammonia lyase (PAL) activity**

Four-week-old *Astragalus* was inoculated with 5 mL *Stenotrophomonas* sp., *Rhizobium* sp., *Ochrobactrum* sp., *Advenella* sp. or four-species community (~ 10^8^ bacteria/mL). Plant samples were collected at 8, 24, 72, 120 and 168 hours after inoculation. Subsequently, 1.0 g plant samples were homogenized in 2.0 ml pre-cooled 0.1 M pH 8.8 sodium borate buffer, containing 5.0 mM of 2-mercaptoethanol and 0.1 g of insoluble polyvinyl pyrrolidine. The homogenate was filtered with 4 layers of gauze and the filtrate was centrifuged at 10,000 g for 15 min. The supernatant was the crude enzyme extract. The PAL activities were determined according to the instructions for plant L-phenylalanine ammonal-lyase (PAL) ELISA Kit (Sinobestbio, Shanghai, China).

**Determination of lipoxygenase (LOX)**

The plant samples (0.5 g) of different inoculation treatments were homogenized in 7 ml of 1 M tris - HCl buffer (Ph = 7.6) in an ice bath. The homogenate was centrifuged at 12000 g/min for 25 minutes. The LOX activities in supernatant was measured using plant LOX ELISA Kit (Sinobestbio, Shanghai, China).

**Determination of chitinase**

Plant samples (0.5 g) of different inoculation treatments were ground into a powder in liquid nitrogen. Added 2 ml 0.05 M phosphate buffer (pH 5.0) to the powder and mixed well. in an ice bath. The mixture was centrifuged at 12,000 g/min for 15 min. Chitinase activities in crude enzyme solution were measured using plant chitinase ELISA Kit (Sinobestbio, Shanghai, China)

**Determination of polyphenol oxidase (PPO)**

The plant samples (1 g) of different inoculation treatments were homogenized in 2 ml of 0.1 M pH = 7.2 citrate phosphate buffer (containing 5% polyvinyl pyrrolidone) in an ice bath. The homogenate was extracted overnight and centrifuged at 12,000 g for 10 min at 4 °C. The supernatant was the crude enzyme extract of PPO. The PPO activities were determined according to manufacturer's instructions for plant polyphenol oxidase (PPO) ELISA Kit (Sinobestbio, Shanghai, China).

**POD activity assay**

Fresh plant (1 g) of different inoculation treatments was homogenized in 5 mL of 0.2 M Tris-buffer (pH 8.0, ontaining 1 g polyvinyl pyrophosphate) and then centrifuged at 1,2000 × g for 15 min. The PPO activities were determined according to manufacturer's instructions for plant peroxidase (POD) ELISA Kit (Sinobestbio, Shanghai, China).

**References:**

[1] Liu D, Chen L, Zhu X, et al. *Klebsiella pneumoniae* SnebYK Mediates Resistance Against *Heterodera glycines* and Promotes Soybean Growth. Frontiers in Microbiology. 2018; 9:1134.

[2] Sheikhian L, Bina S. Simultaneous extraction and HPLC determination of 3-indole butyric acid and 3-indole acetic acid in pea plant by using ionic liquid-modified silica as sorbent. Journal of Chromatography B. 2016; 1009-1010:34-43.

[3] Fang H, Luo F, Li P, Zhou Q, Zhou X, Wei B, Cheng S, Zhou H, Ji S. Potential of jasmonic acid (JA) in accelerating postharvest yellowing of broccoli by promoting its chlorophyll degradation. Food Chem. 2020;309:125737.


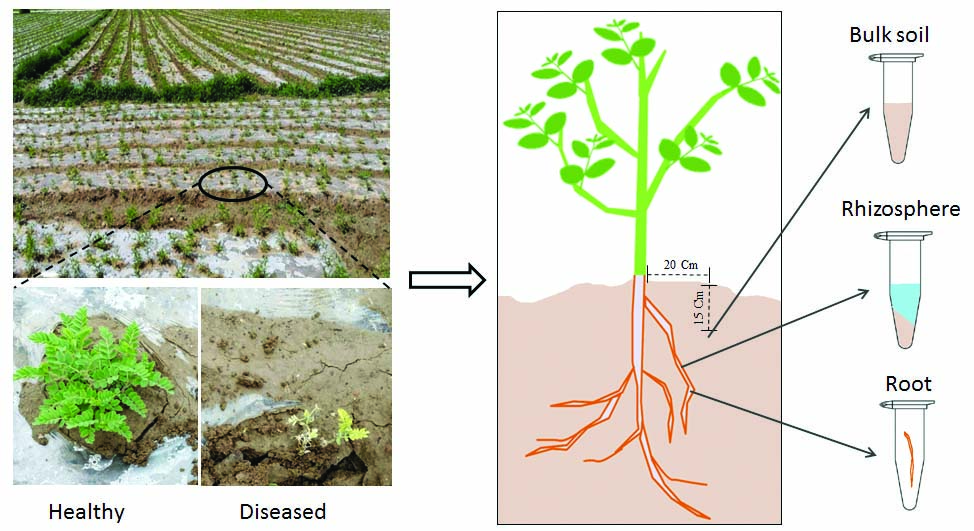


**Fig. s1 Schematic diagram of root and soil sample collection.** Twelve healthy and twelve diseased *Astragalus* were randomly selected from the field. Bulk soil was sampled 20cm from the principal root at depths of 15cm with a soil corer, while the roots were collected by extracting individual plants with a shovel. Roots were shaken vigorously to remove loose soil and the rhizosphere soil was eluted from roots with 1× phosphate buffer.

**
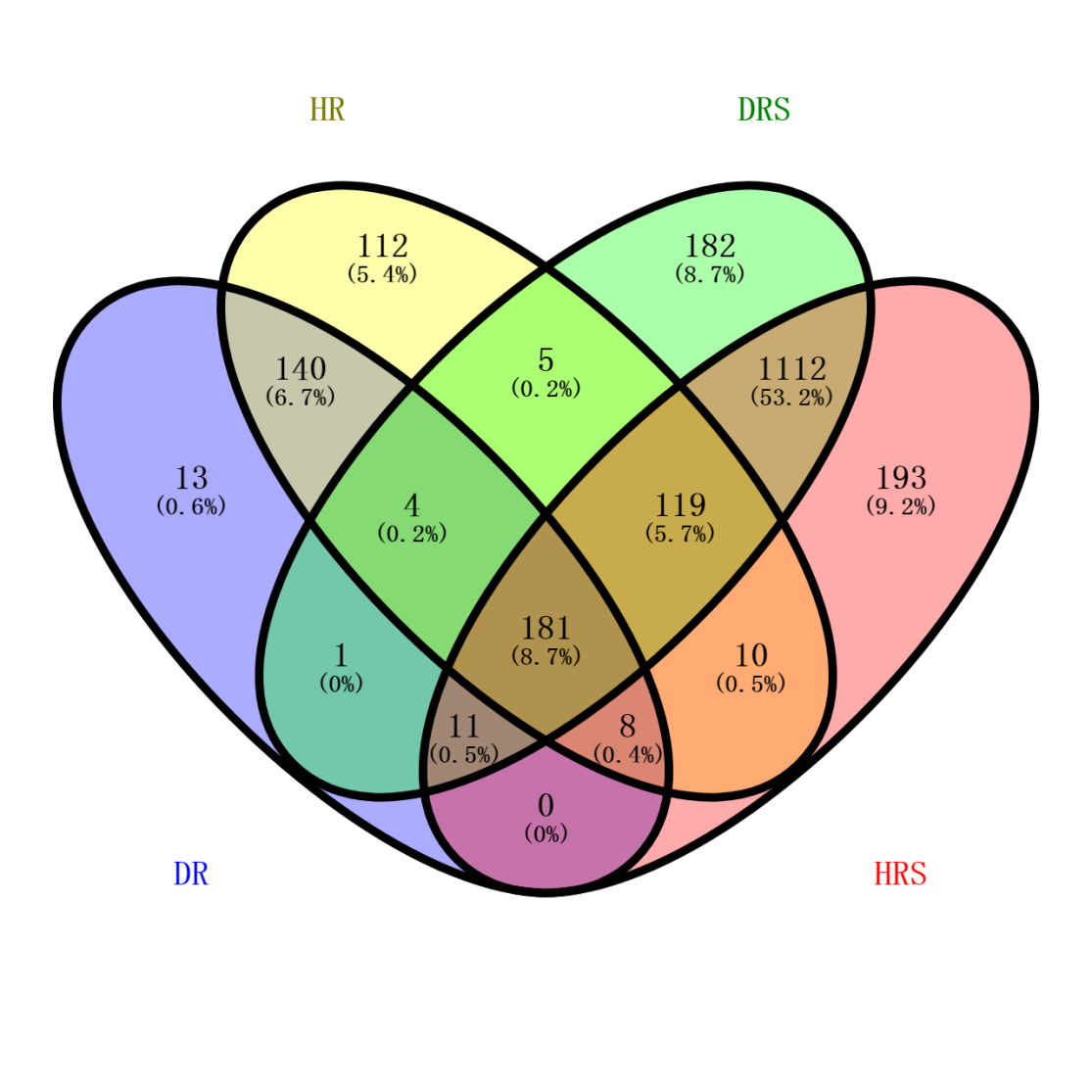
Fig. s2 The distinct and shared bacterial genera in different sample compartment.** All the soil and plant samples were collected from three medicinal plant fields at Tanchang county, China. Four healthy and four diseased plants were randomly collected from each field. The distinct and shared bacteria were analyzed in Venn diagrams using ‘VennDiagram’ package in R V3.6.0. HRS: Healthy rhizosphere; DRS: Diseased rhizosphere; HR: Healthy root; DR: Diseased root.


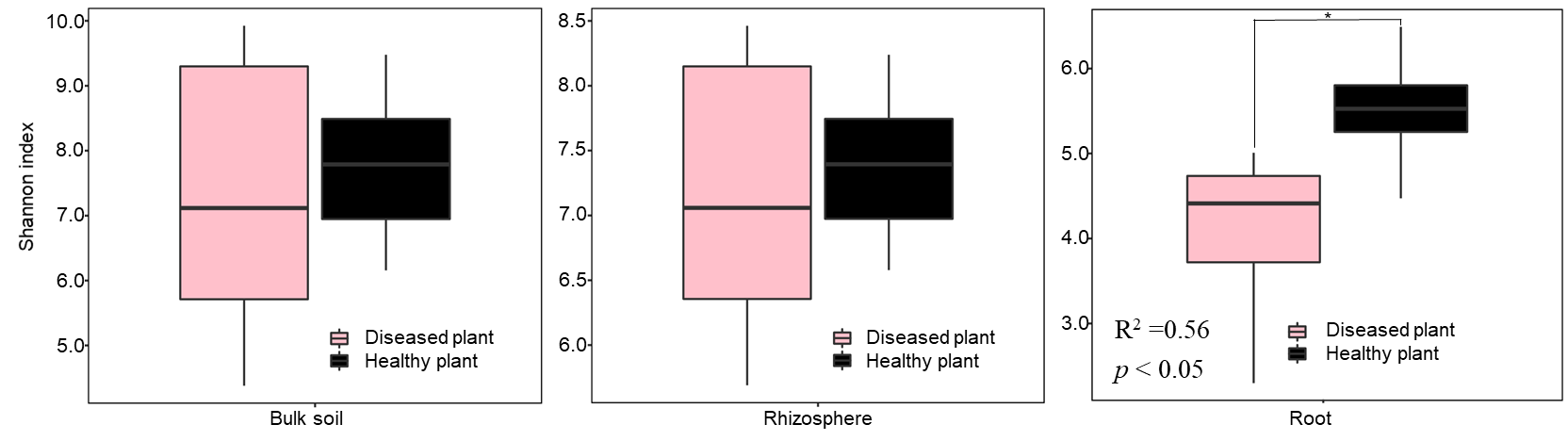


**Fig. s3** **Effect of fungal pathogen on Shannon diversity of bacterial communities in bulk soil, rhizosphere and root**. *F. oxysporum* decreased the Shannon alpha diversity of bacterial communities in roots but had no effect on alpha diversity of bacterial communities in bulk soil and rhizosphere. Asterisks indicate statistically significant (*, *P* < 0.05) differences based on analysis of variance with Dunn’s multiple comparison test.


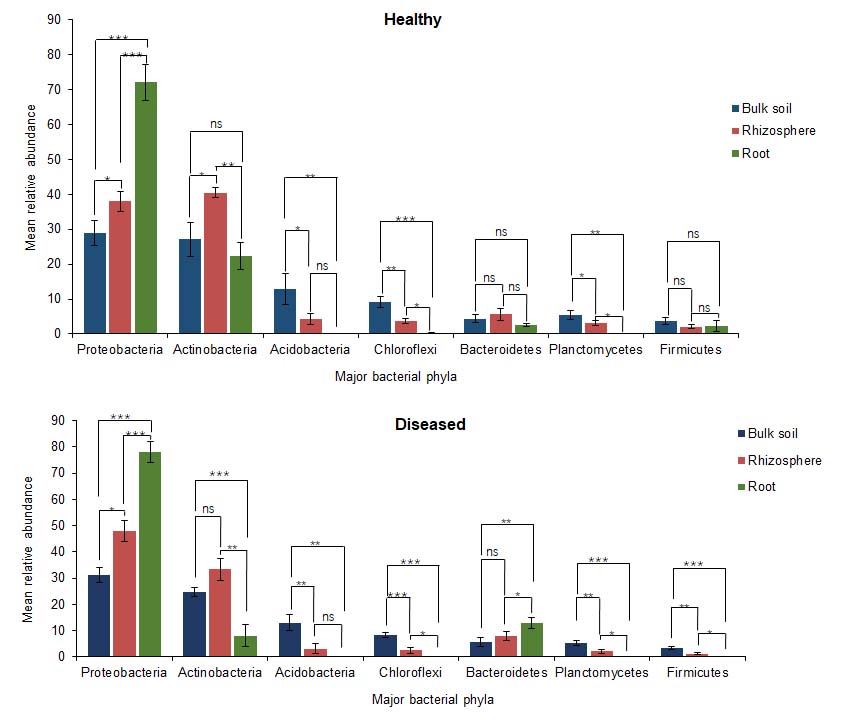


**Fig. s4** **Enrichment of bacterial phyla by** ***Astragalus* roots**. The graph above showed the abundance of major bacterial phyla in different compartments of healthy plants. Healthy rhizosphere significantly enriched Proteobacteria and Actinobacteria, while healthy roots enriched Proteobacteria. The graph below showed the abundance of major phyla in different compartments of diseased plants. Diseased rhizosphere enriched Proteobacteria, while diseased roots enriched Proteobacteria and Bacteroidetes. Blue, red and green bars indicated bulk soil, rhizosphere and roots. To determine whether relative abundance of bacterial phyla differed among bulk soil, rhizospheres and roots, an ANOVA was performed. Significant differences between compartments are indicated with asterisks above columns (*, *p* < 0.05; **, *p* < 0.01; ***, *p* < 0.0001).


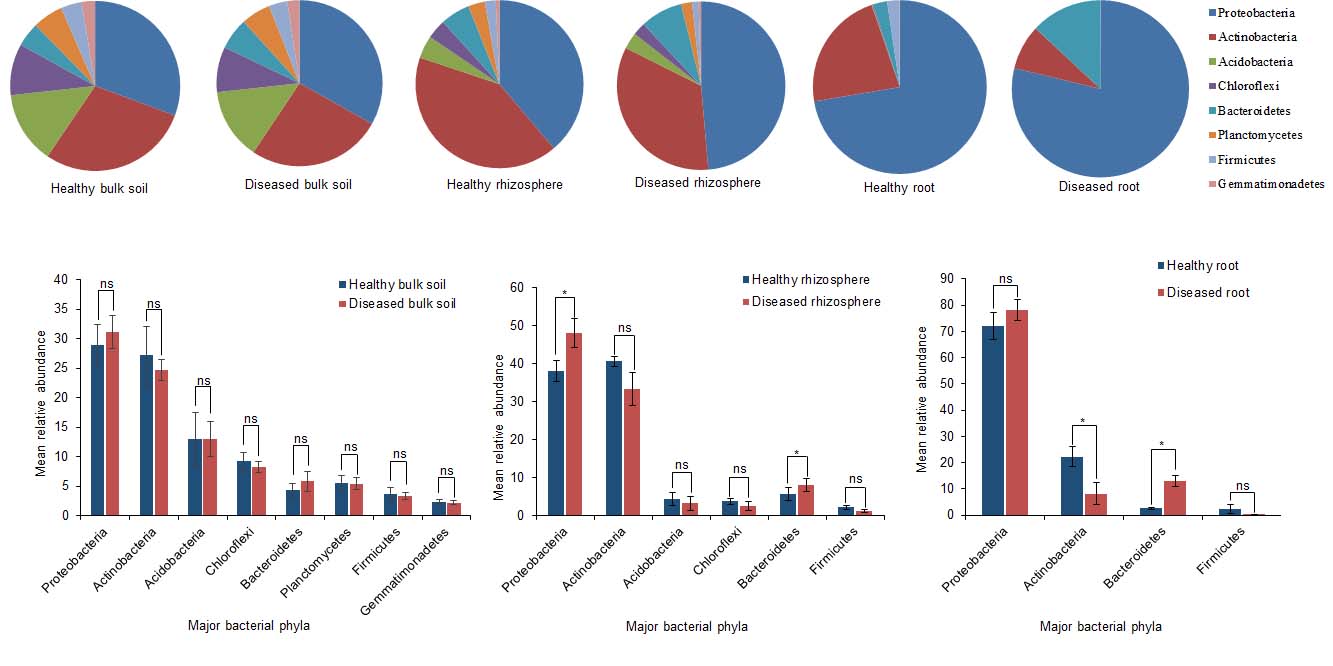


**Fig. s5** **Effect of *F. oxysporum* infection on the abundance of major bacterial phyla**. (A) The change of the proportions of phyla in different compartments under the pathogenic fungal infection. (B) *Fusarium oxysporum* did not affect the abundance of major bacterial phyla (left) in bulk soil but affect the abundance of Proteobacteria and Bacteroidetes in rhizosphere (middle), and Actinobacteria and Bacteroidetes in roots (right). Blue and red bars indicated healthy and diseased samples. To determine if relative abundance of bacterial phyla differed between healthy and diseased samples, Tukey’s post-hoc test was performed. Significant differences are indicated with asterisks above columns (*, *p* < 0.05).


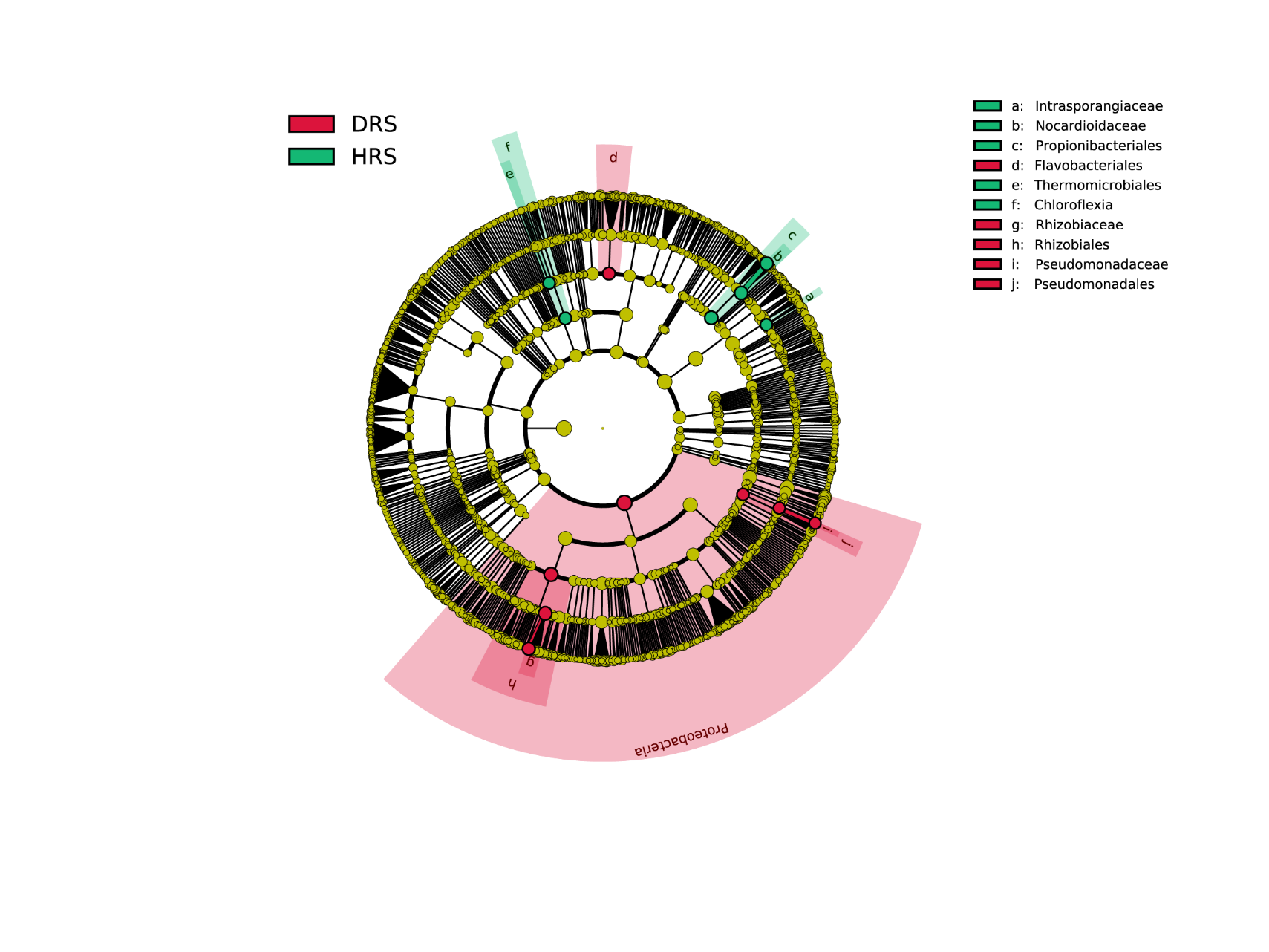


**Fig. s6 Least discriminant analysis (LDA) effect size taxonomic cladogram comparing healthy and diseased rhizosphere samples.** The different classification levels are represented from inside to outside. The red nodes indicate enriched-genera in diseased rhizosphere, yellow nodes indicate no difference, and green nodes indicate depleted-genera in diseased rhizosphere. HRS: Healthy rhizosphere, HR: Healthy root.


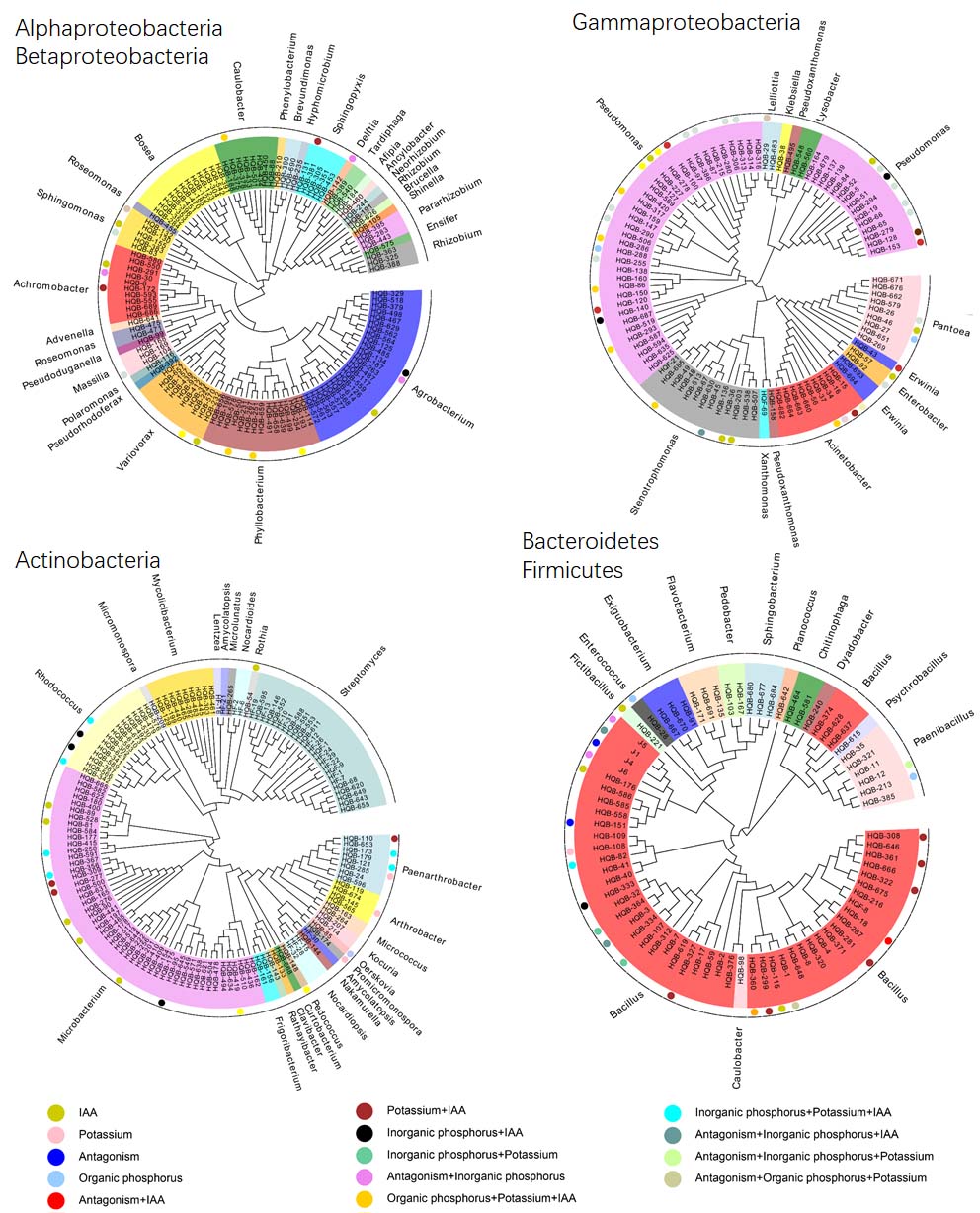


**Fig. s7 Plant growth-promoting properties of plant-associated bacteria.** Bacterial strains were isolated from healthy and diseased rhizosphere or roots. Neighbour-joining tree generated according to the 16S rRNA gene sequences of 423 bacterial strains. Bacteria shaded in different colors indicate which genus the strains belong to. The circles with different color in outer ring showed that the strain has one or more functions of indole acetic acid (IAA) production, inorganic phosphorus dissolution, organic phosphorus dissolution, potash feldspar dissolution, and antagonism.


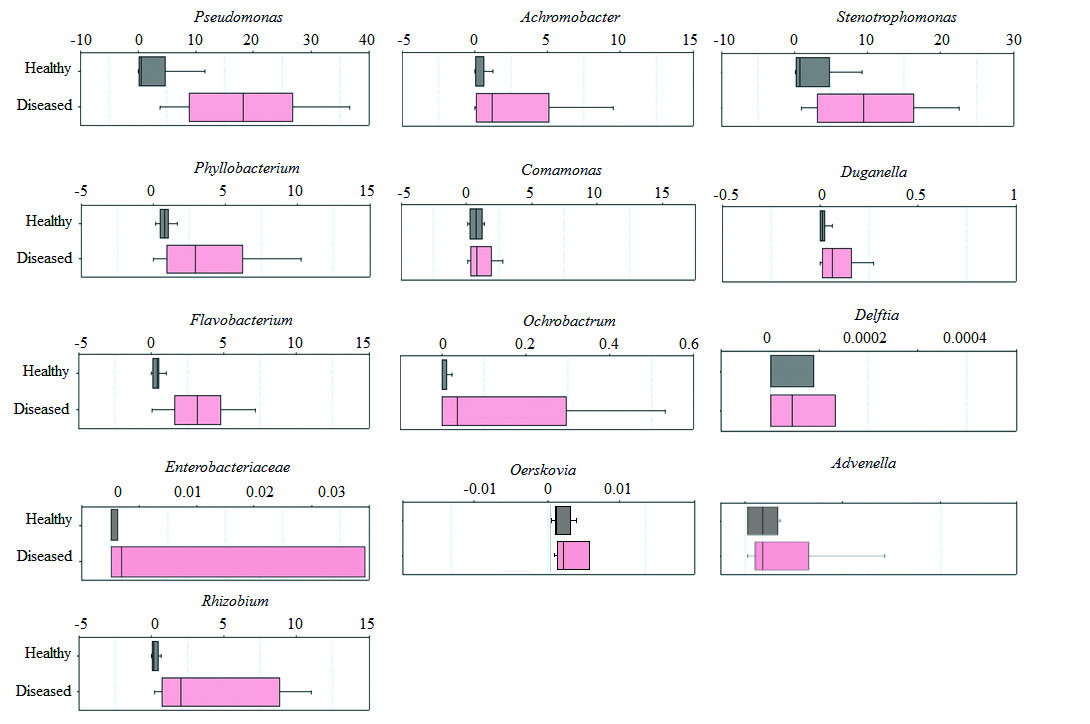


**Fig. s8 Relative abundance of bacteria in SCI found in healthy and diseased roots.** Gray and pink columns represent the relative abundance of bacterial genera in healthy and diseased roots. The abundance of *Pseudomonas*, *Stenotrophomonas*, *Phyllobacterium* and *Flavobacterium* in diseased roots increased more than that of other strains.


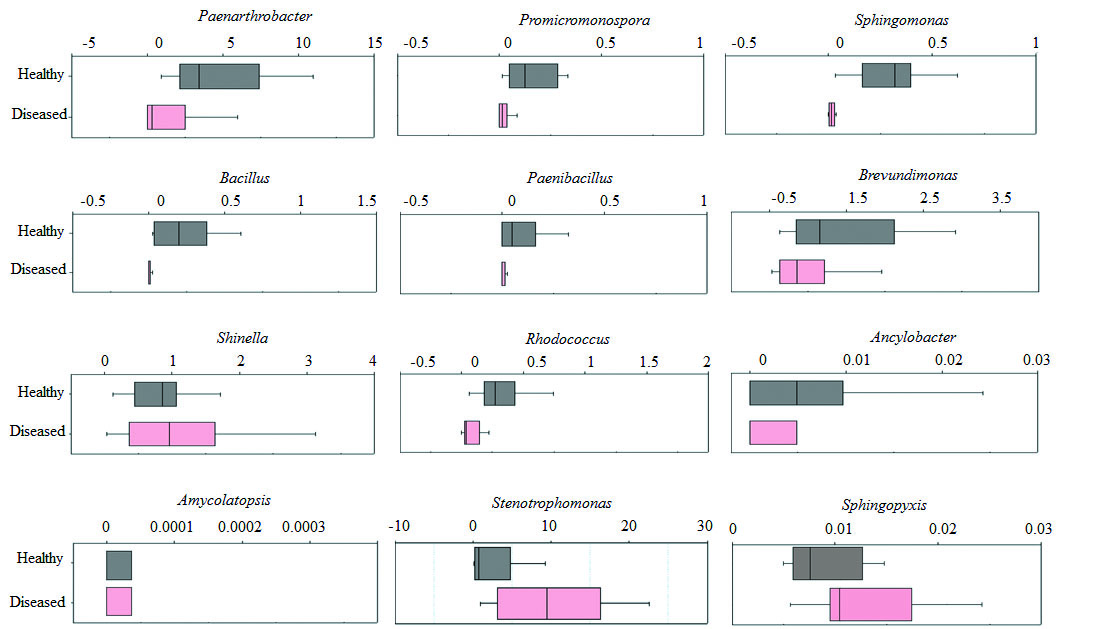


**Fig. s9 Relative abundance of bacteria in SCII found in healthy and diseased roots.** Gray and pink columns represent the relative abundance of bacterial genera in healthy and diseased roots. Abundance of nine strains (*Paenarthrobacter*, *Promicromonospora*, *Spingomonas*, *Bacillus*, *Paenibacillus*, *Brevundimonas*, *Rhodococcus* and *Ancylobacter*; Two bacterial species were selected from *Bacillus* genera) in diseased roots was decreased, while five bacterial strains (*Shinella*, *Amycolatopsis*, *Stenotrophomonas* and *Sphingopyxis*) were randomly selected to assemble SCII. The abundance of *Paenarthrobacter*, *Brevundimonas* and *Bacillus* in diseased roots decreased more than that of other strains.


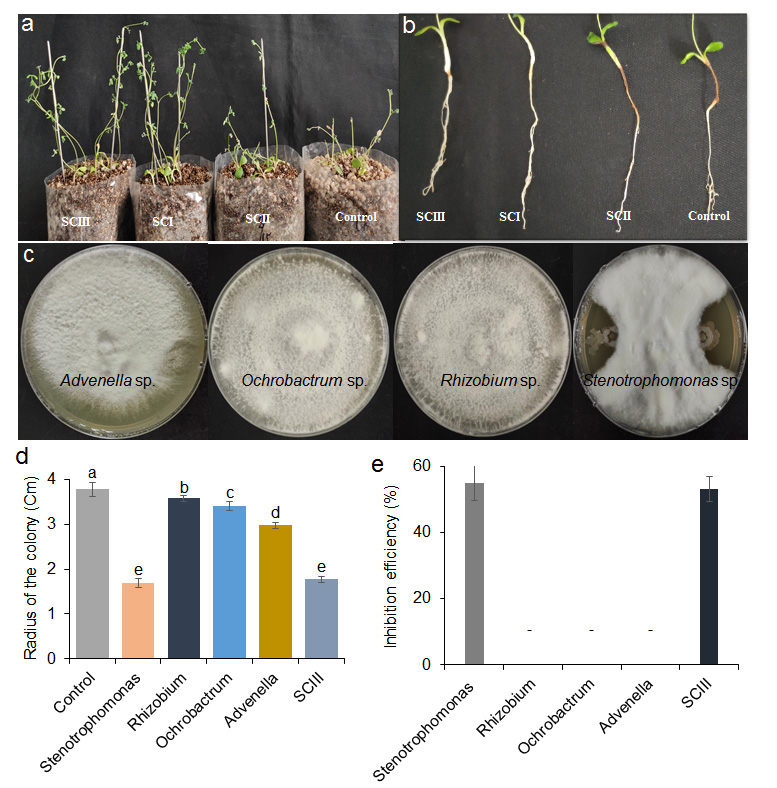


**Fig. s10 Inhibition of four bacteria in simple synthetic community (SCIII) on the growth of *F. oxysporum*.** (a) Shoot phenotype of plants inoculated with different bacterial communities and *F. oxysporum*. SCI: synthetic community I; SCII: synthetic community II; SCIII: four-species simple community (b) Colony radii of *F. oxysporum* treated with different bacteria after 6 d of growth (n = 3). (c) Mycelium growth inhibition efficiency. *Stenotrophomonas* sp. and four-species community exhibits relatively high inhibitory efficiency, while *Rhizobium* sp., *Advenella* sp., and *Ochrobactrum* sp. has minimal effect on *F. oxysporum* growth after 10 d. Letters indicate significant differences (*p* < 0.05). (d) The effect of four bacterial strains on *F. oxysporum* growth after 10 d in Potato Dextrose Agar plates.


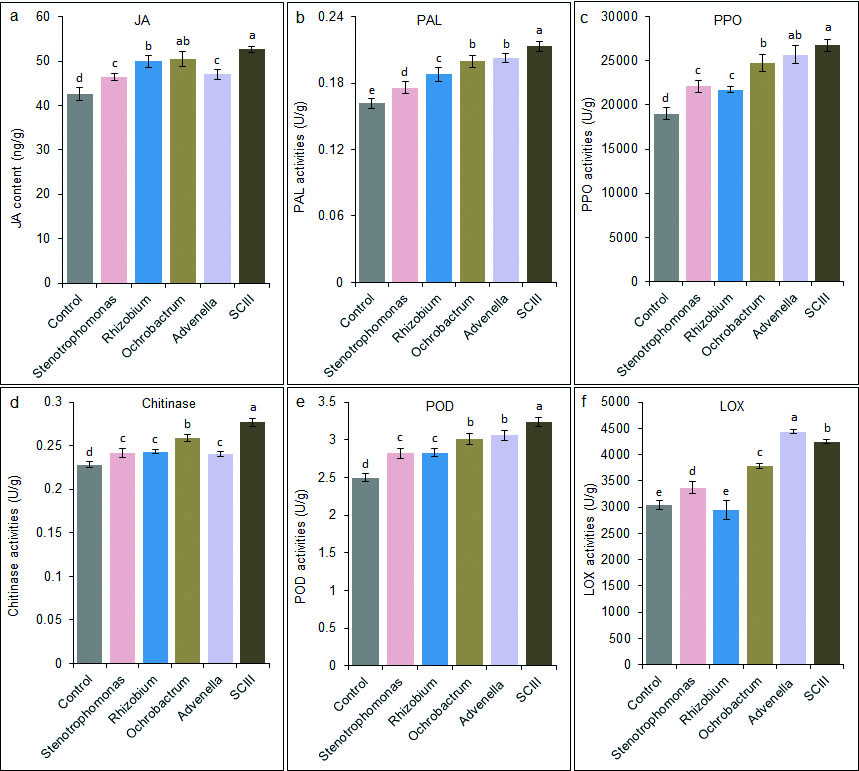


**Fig. s11** **Effect of SCIII and single bacterium inoculation on the ISR of *Astragalus***. JA content and PAL, POD, PPO, LOX, and chitinase activity of plants on the 7^th^ day after inoculation with sterile water, *Stenotrophomonas* sp., *Rhizobium* sp., *Ochrobactrum* sp., *Advenella* sp. and SCIII, respectively. The letters denote differences based on Duncan's multiple range test (n = 3, *p* < 0.05). JA, jasmonic acid; PAL, phenylalanine ammonia lyase; PPO, polyphenol oxidase; POD, peroxidase; LOX, lipoxygenase; ISR, induced systemic resistance; SCIII, four species synthetic community.
