# Supplementary material for: A simplified synthetic community rescues Astragalus mongholicus from root rot disease by activating plant-induced systemic resistance
Source: Microbiome. 2021 Nov 4;9:217. doi: 10.1186/s40168-021-01169-9 (PMC8567675; doi:10.1186/s40168-021-01169-9)
Supplement: Supplementary file 3 — Additional file 2: Table S1. Characteristics of bacteria in two synthetic communities. [file 40168_2021_1169_MOESM3_ESM.docx]

| Table s1 Characteristics of bacteria in two synthetic communities | | | | | | |  |
| --- | --- | --- | --- | --- | --- | --- | --- |
|  | Strain | Antagonism to  *F. oxysporum* | Produing IAA | Inorganic phosphorus dissolve | Organic phosphorus dissolve | Potash feldspar dissolve | Sources |
| Sythetic community 1 | *Pseudomonas* sp. |  | + | + | + |  | Diseased root |
|  | *Achromobacter* sp. | + |  | + |  |  | Diseased root |
|  | *Delftia* sp.* | + |  | + |  |  | Healthy root |
|  | *Enterobacter* sp.* | + |  | + | + | + | Healthy root |
|  | *Advenella* sp.* |  |  |  |  |  | Healthy Rhizosphere |
|  | *Flavobacterium* sp. |  |  |  |  |  | Healthy Rhizosphere |
|  | *Duganella* sp. |  |  |  |  |  | Healthy Rhizosphere |
|  | *Stenotrophomonas* sp. | + | + | + |  |  | Diseased root |
|  | *Ochrobactrum* sp. |  |  |  |  |  | Healthy root |
|  | *Phyllobacterium* sp. |  |  | + | + |  | Diseased root |
|  | *Comamonas* sp. |  | + |  |  | + | Diseased root |
|  | *Oerskovia* sp. |  |  |  | + | + | Healthy Rhizosphere |
|  | *Rhizobium* sp. |  |  | + |  |  | Diseased root |
| Sythetic community 2 | *Paenarthrobacter* sp. |  |  |  |  |  | Healthy root |
|  | *Promicromonospora* sp.* |  |  |  |  | + | Healthy Rhizosphere |
|  | *Sphingomonas* sp. | + |  | + | + |  | Healthy root |
|  | *Ancylobacter* sp.*** |  |  |  |  |  | Diseased root |
|  | *Bacillus* sp. | + | + | + |  |  | Healthy root |
|  | *Bacillus* sp. |  |  |  |  |  | Diseased root |
|  | *Paenibacillus* sp. | + |  | + |  | + | Healthy root |
|  | *Brevundimonas* sp. |  |  |  |  |  | Healthy root |
|  | *Shinella* sp. |  |  |  |  |  | Healthy Rhizosphere |
|  | *Rhodococcus* sp.* |  |  |  |  |  | Diseased root |
|  | *Sphingopxis* sp. |  | + |  |  | + | Diseased root |
|  | *Stenotrophomonas* sp. |  |  |  |  |  | Healthy root |
|  | *Amycolatopsis* sp.*** | + |  |  |  |  | Healthy Rhizosphere |

Note: The synthetic community 1 was assembled with genera decreased in diseased roots. One species was selected for each genus, and the strains with plant growth promoting characteristics were preferred to select. The synthetic community 2 was constructed with nine depleted and randomly four genera in diseased roots. All the bacterial strains used to construct the communities were identified by 16S rRNA sequencing. The asterisked strains indicate a relative abundance of this genus less than 0.1%
