# Supplementary material for: A simplified synthetic community rescues Astragalus mongholicus from root rot disease by activating plant-induced systemic resistance
Source: Microbiome. 2021 Nov 4;9:217. doi: 10.1186/s40168-021-01169-9 (PMC8567675; doi:10.1186/s40168-021-01169-9)
Supplement: Supplementary file 4 — Additional file 3: Table S2. Diversity index of bacterial community in different compartment of A. mongholicus. [file 40168_2021_1169_MOESM4_ESM.docx]

Table s2 Diversity index of bacterial community in different compartment of *A. mongholicus*

|  | Richness | Shannon | Simpson | Pielou | Chao1 | ACE |
| --- | --- | --- | --- | --- | --- | --- |
| Bulk soil of healthy plant | 2203.25 ± 156.63^a^ | 9.18 ± 0.39^a^ | 0.99 ± 0.004^a^ | 0.83 ± 0.029^a^ | 92.62 ± 8.63^a^ | 30.77 ± 1.13^a^ |
| Bulk soil of diseased plant | 2154.69 ± 408.82^a^ | 9.08 ± 0.79^a^ | 0.99 ± 0.006^a^ | 0.82 ± 0.049^a^ | 94.79 ± 8.97^a^ | 30.38 ± 2.20^a^ |
| Rhizosphere of healthy plant | 1499.13 ± 279.83^b^ | 7.41 ± 0.64^b^ | 0.97 ± 0.013^a^ | 0.70 ± 0.045^b^ | 90.75 ± 7.25^a^ | 26.98 ± 1.78^b^ |
| Rhizosphere of diseased plant | 1397.5 ± 377.52^b^ | 7.06 ± 0.97^b^ | 0.96 ± 0.035^a^ | 0.68 ± 0.073^b^ | 90.32 ± 8.91^a^ | 26.74 ± 2.93^b^ |
| Root of healthy plant | 286.69 ± 70.07^c^ | 5.33 ± 0.85^c^ | 0.92 ± 0.064^b^ | 0.65 ± 0.085^bc^ | 28.93 ± 12.40^b^ | 9.98 ± 1.03^c^ |
| Root of diseased plant | 168.94 ± 56.96^d^ | 4.27 ± 0.81^d^ | 0.86 ± 0.096^c^ | 0.58 ± 0.079^c^ | 22.80 ± 8.01^b^ | 8.16 ± 1.72^d^ |

Note: The effects of *F. oxysproum* infection and nich on bacterial alpha diversity indices were calculated using ‘vegan’ pakage in an R v3.6.0 environment

(R Software Foundation, Vienna, Austria). Letters indicate significant differences *P* < 0.05.
